# Molecular Mean-Field Theory of Ionic Solutions: A Poisson-Nernst-Planck-Bikerman Model

**DOI:** 10.3390/e22050550

**Published:** 2020-05-14

**Authors:** Jinn-Liang Liu, Bob Eisenberg

**Affiliations:** 1 Institute of Computational and Modeling Science, National Tsing Hua University, Hsinchu 300, Taiwan; 2 Department of Physiology and Biophysics, Rush University, Chicago, IL 60612, USA; beisenbe@rush.edu; 3 Department of Applied Mathematics, Illinois Institute of Technology, Chicago, IL 60616, USA

**Keywords:** bioelectricity, electrochemistry, thermodynamics, electrokinetics, molecular mean-field theory, Boltzmann and Fermi distributions, Poisson–Boltzmann, Poisson–Fermi, Poisson–Bikerman, Nernst–Planck, steric and correlation effects, ion channels, ion activity, double-layer capacitance, nanofluidics

## Abstract

We have developed a molecular mean-field theory—fourth-order Poisson–Nernst–Planck–Bikerman theory—for modeling ionic and water flows in biological ion channels by treating ions and water molecules of any volume and shape with interstitial voids, polarization of water, and ion-ion and ion-water correlations. The theory can also be used to study thermodynamic and electrokinetic properties of electrolyte solutions in batteries, fuel cells, nanopores, porous media including cement, geothermal brines, the oceanic system, etc. The theory can compute electric and steric energies from all atoms in a protein and all ions and water molecules in a channel pore while keeping electrolyte solutions in the extra- and intracellular baths as a continuum dielectric medium with complex properties that mimic experimental data. The theory has been verified with experiments and molecular dynamics data from the gramicidin A channel, L-type calcium channel, potassium channel, and sodium/calcium exchanger with real structures from the Protein Data Bank. It was also verified with the experimental or Monte Carlo data of electric double-layer differential capacitance and ion activities in aqueous electrolyte solutions. We give an in-depth review of the literature about the most novel properties of the theory, namely Fermi distributions of water and ions as classical particles with excluded volumes and dynamic correlations that depend on salt concentration, composition, temperature, pressure, far-field boundary conditions etc. in a complex and complicated way as reported in a wide range of experiments. The dynamic correlations are self-consistent output functions from a fourth-order differential operator that describes ion-ion and ion-water correlations, the dielectric response (permittivity) of ionic solutions, and the polarization of water molecules with a single correlation length parameter.

## 1. Introduction

Water and ions give life. Their electrostatic and kinetic interactions play essential roles in biological and chemical systems such as DNA, proteins, ion channels, cell membranes, physiology, nanopores, supercapacitors, lithium dendrite growth, porous media, corrosion, geothermal brines, environmental applications, and the oceanic system [1,2,3,4,5,6,7,8,9,10,11,12,13,14,15,16,17,18,19,20,21,22,23,24,25,26,27,28,29,30,31,32,33,34]. Poisson, Boltzmann, Nernst, and Planck laid the foundations of classical electrostatic and kinetic theories of ions in 1813–1890 [35,36,37,38,39]. Gouy [40] and Chapman [41] formulated the Poisson–Boltzmann (PB) equation in 1910 and 1913, respectively [9]. Bikerman proposed a modified PB equation in 1942 for binary ionic liquids to account for **different-sized** ions with **voids** [42]. Eisenberg puns PNP for Poisson-Nernst-Planck and Positive-Negative-Positive semiconductor transistors to emphasize nonequilibrium flows of ions through ion channels as life’s transistors [43]. Ions in classical PB and PNP theories are treated as volumeless point charges like the ‘ions’ of semiconductors, namely holes and electrons in semiconductor electronics [44,45,46,47,48,49,50,51,52,53,54,55]. Water molecules are treated as a dielectric medium (constant) without volumes either. However, advanced technologies in ion channel experiments [56,57] and material science [33,34,58] have raised many challenges for classical continuum theories to describe molecular mechanisms of ions and water (or solvents) with specific size effects in these systems at nano or atomic scale [9,12,14,16,17,18,19,30,33].

There is another important property that classical continuum theories fail to describe, namely short-range **ion-ion** or **ion-water correlations** in ion channels [8,9], charge-induced thickening and density oscillations near highly charged surfaces [14], correlation-induced charge inversion on macroions (DNA, actin, lipid membranes, colloidal particles) [59], the phase structure of plasma and polar fluids [60], colloidal charge renormalization [60], etc. Several other properties related to correlations such as the dielectric response of electrolytes solutions and the polarization of water in various conditions or external fields are usually modeled differently from the correlation perspective [61,62,63].

We have recently developed a molecular mean-field theory called—Poisson-Nernst-Planck-Bikerman (PNPB) theory—that can describe the size, correlation, **dielectric**, and **polarization** effects of ions and water in aqueous electrolytes at equilibrium or nonequilibrium all within a unified framework [64,65,66,67,68,69,70,71,72,73,74,75,76]. Water and ions in this theory can have *different shapes* and *volumes* necessarily with intermolecular voids. The theory generalizes and unifies the second-order Poisson–Bikerman equation [42] of binary ionic liquids for different-sized ions with identical steric energies [72] and the **fourth-order** differential permittivity operator in Santangelo’s model of one component plasma [77] or in the Bazant, Storey, and Kornyshev theory of general nonlocal permittivity for equal-sized ions in ionic liquids [78].

Ion-ion and ion-water correlations are modeled by the permittivity operator with a correlation length that depends on the diameter of ions or water and the valence of ions of interest [78]. The fourth-order operator yields a permittivity as an *output function* of spatial variables, salt concentration, and hydration shell structure including water diameter from solving the PNPB model and thus describes the **dehydration** of ions from bath to channel pore or from bulk to charged wall, the polarization of water, and the change of permittivities of electrolyte solutions at different locations in response to different configurations and conditions. Water densities also change with configurations and conditions.

The fourth-order operator introduces correlations into the mean-field equations so they can deal more realistically with real systems in which the correlations are of the greatest importance. A remark should be made here that simulations containing only particles do not automatically deal with correlations better than mean-field theories with fourth-order operators like this. It is not at all clear that simulations widely done in biophysics actually compute correlations well. Indeed, it is difficult to see how simulations that use conventions to approximate the electric field, and periodic boundary conditions to approximate macroscopic systems could deal with correlations correctly. The dearth of direct checks of the role of periodic boundary conditions, and of the accuracy of the conventional treatment of electrostatics, does little to assuage these concerns. The detailed direct checks found necessary in computational electronics are not easily found in simulations of ions in electrolyte solutions (see Chap. 6, particularly Figures 6.34–35 of [55] for some details that are found to be necessary in the simulations of computational electronics).

It is important to reiterate the obvious. Our model includes water as a molecule and depends on the hydration structure around ions. Our model uses partial differential equations (PDEs) to describe these essentially discrete properties of ionic solutions, and uses the physical parameters of individual atoms and water molecules, NOT just their mean-field description. This use of PDEs to describe inherently discrete processes is hardly new: most of probability theory [79,80] and the entire theory of wave equations, including the wave equation of the electron called the Schrödinger equation [81], treat discrete processes the same way, using PDEs that measure (in probability theory) the underlying discrete system, or represent it exactly as the discrete solutions of a continuum PDE (e.g., the Schrödinger equation describing a hydrogen atom).

The most important contribution of our work is to include water as discrete molecules by using **Fermi distributions** [82] of classical particles having excluded volumes with interstitial voids. We show that the treatment of water as finite size molecules requires, as a matter of mathematics, not physics, the existence of voids. This is demonstrated by mathematics and simple ways to compute the voids and their role are presented. These Fermi-like distributions yield **saturation** of all particles (ions and water) even under mathematically infinite large external fields. These distributions also satisfy **mass conservation** in the region of interest such as channel pores, which classical theories fail to describe as well. This Fermi distribution of classical particles obeying volume exclusion is reminiscent of the Fermi distribution of identical particles obeying the Pauli exclusion principle [83] in quantum mechanics.

We also introduce a new concept of distance-dependent potential between non-bonded particles for different-sized particles similar to the electric potential for different-charged ions and name it the **steric potential**. The void distribution function describes the van der Waals potential [84] of paired particles [85,86] in the system in a mean-field sense. The steric potential can be written as a distribution function of voids, emphasizing the crucial role of voids in our theory. The specific sizes of particles and the distance-dependent steric potential allow us to calculate steric energies at the **atomic scale**. Using Coulomb’s law allows calculation of electric energies at the atomic scale as well. Therefore, our theory applies to biological or chemical systems with explicit atomic structures, as well as classical mean-field representations of bulk solutions, for example. We have shown that solving the PNPB model in different continuum and molecular domains yields *self-consistent* electric and steric potentials in many examples of biological ion channels or chemical systems in [64,65,66,67,68,69,70,71,72,73,74,75,76]. The theory is also *consistent* with classical theories in the sense that its model converges to the corresponding classical one when the volume of all particles and the correlation length tend to zero, i.e., steric and correlation effects vanish asymptotically to classical cases.

In this review article, we explain the above bold-face terms in detail and compare them with those of earlier theories in a precise but limited way. The precision means that we display explicitly, to the best of our ability, the significant differences between analogous concepts in our theory and previous treatments. It is obviously impossible to do complete comparisons in this vast and formidable field. No doubt we are ignorant of significant relevant papers. We apologize to those inadvertently slighted and ask them to help us remedy our oversight. The remaining of this article consists as follows.

Section 2 describes the physical meaning of Fermi distributions and the steric potential of ions and water with excluded volumes. We also explain the differences between Fermi and Boltzmann distributions in the context of statistical thermodynamics.

Section 3 unifies Fermi distributions and correlations into the simple and concise 4th-order Poisson–Bikerman (4PBik) equation. The simplicity refers to the correlation length being the only empirical parameter in the equation. The conciseness means that the fourth-order differential operator can describe the complex and correlated properties of ion-ion and ion-water interactions, polarization of water, and dielectric response of electrolytes solutions all in a single model setting.

Section 4 presents a Gibbs free energy functional for the 4PBik equation. We show that minimization of the functional yields the equation and Fermi distributions that reduce to Boltzmann distributions when the volumes of particles vanish in limiting case. This functional is critical to explain a major shortcoming of earlier modified PB models that cannot yield Boltzmann distributions in the limit. These models are thus not consistent with classical theories and may poorly estimate steric energies and other physical properties due to their coarse approximation of size effects.

Section 5 generalizes the 4PBik equation to the PNPB model to describe flow dynamics of ions and water in the system subject to external fields. The most important feature in this section is the introduction of the steric potential to the classical Nernst–Planck equation. Electric and steric potentials describe the dynamic **charge/space competition** between ions and water. We also show that the PNPB model reduces to the 4PBik equation at equilibrium.

Section 6 presents a generalized Debye-Hückel theory from the 4PBik equation for thermodynamic modeling. The theory yields an equation of state that analytically models ion activities in all types of binary and multi-component electrolyte solutions over wide ranges of concentration, temperature, and pressure. It is also useful to study the size, correlation, dielectric, and polarization effects in a clear comparison with those ignoring these effects.

Section 7 discusses numerical methods for solving the PNPB model that is highly nonlinear and complex when coupled with the electrical field generated by protein charges in ion channels, for example. It is very challenging to numerically solve the model with tolerable accuracy in 3D protein structures that generate extremely large electric field, e.g., 0.1 V in 1 Angstrom, in parts of the molecule of great biological importance where crowded charges directly control biological function, in the same sense that a gas pedal controls the speed of a car.

Section 8 demonstrates the usefulness of the PNPB theory for a wide range of biological and chemical systems, where the steric and correlation effects are of importance. We choose a few examples of these systems, namely electric double layers, ion activities, and biological ion channels.

Section 9 summarizes this review with some concluding remarks.

## 2. Fermi Distributions and Steric Potential

The total volume of an aqueous electrolyte system with *K* species of ions in a solvent domain $\Omega_{s}$ is

(1) $$V=\sum_{i=1}^{K+1}v_{i}N_{i}+V_{K+2},$$

where K+1 and K+2 denote water and voids, respectively, $v_{i}$ is the volume of each species *i* particle, $N_{i}$ is the total number of species *i* particles, and $V_{K+2}$ is the total volume of all the voids [68]. The volume of each particle $v_{i}$ will play a central role in our analysis, as well that the limit $v_{i}$ goes to zero. This limit defines the solution of point particles of classical PB and PNP theory. We must include the voids as a separate species if we treat ions and water having volumes in a model. This necessity can be proven by mathematics (see below). It is also apparent to any who try to compute a model of this type with molecular water, as it was to us [68].

Dividing the volume Equation (1) in bulk conditions by *V*, we get the bulk volume fraction of voids

(2) $$\Gamma^{B}=1-\sum_{i=1}^{K+1}v_{i}C_{i}^{B}=\frac{V_{K+2}}{V},$$

where $C_{i}^{B}=\frac{N_{i}}{V}$ are bulk concentrations. If the system is spatially inhomogeneous with variable electric or steric fields, as in realistic systems, the constants $C_{i}^{B}$ then change to functions $C_{i}\left(r\right)$ and so does $\Gamma^{B}$ to a void volume function

(3) $$\Gamma \left(r\right)=1-\sum_{i=1}^{K+1}v_{i}C_{i}\left(r\right).$$

We define the concentrations of particles (i.e., the distribution functions of the number density) in $\Omega_{s}$ [72] as

(4) $$C_{i}\left(r\right)=C_{i}^{B}\exp\left(-\beta_{i}\phi \left(r\right)+\frac{v_{i}}{v_{0}}S^{trc}\left(r\right)\right),\;\;S^{trc}\left(r\right)=\ln\frac{\Gamma (r)}{\Gamma^{B}},$$

where ϕ(r) is an electric potential, $S^{trc}\left(r\right)$ is called a *steric potential*, $\beta_{i}=q_{i}/k_{B}T$ with $q_{i}$ being the charge on species *i* particles and $q_{K+1}=0$, $k_{B}$ is the Boltzmann constant, *T* is an absolute temperature, and $v_{0}=\left(\sum_{i=1}^{K+1}v_{i}\right)/\left(K+1\right)$ is an average volume. The following inequalities

(5) $$\begin{array}{ccc} C_{i}\left(r\right) & = & C_{i}^{B}\exp\left(-\beta_{i}\phi \left(r\right)\right){\left[\frac{\Gamma (r)}{\Gamma^{B}}\right]}^{v_{i}/v_{0}}=\alpha_{i}{\left[1-\sum_{j=1}^{K+1}v_{j}C_{j}\left(r\right)\right]}^{v_{i}/v_{0}}\\ & = & \alpha_{i}{\left[1-v_{i}C_{i}\left(r\right)-\sum_{j=1,j\neq i}^{K+1}v_{j}C_{j}\left(r\right)\right]}^{v_{i}/v_{0}}<\alpha_{i}{\left[1-v_{i}C_{i}\left(r\right)\right]}^{v_{i}/v_{0}}\\ & \leq & \alpha_{i}\left[1-\frac{v_{i}^{2}}{v_{0}}C_{i}\left(r\right)\right]\;\mathrm{if}\;v_{i}/v_{0}\leq 1,\;\mathrm{by}\;\mathrm{Bernoulli}’s\;\mathrm{inequality}, \end{array}$$

(6) $$\begin{array}{ccc} C_{i}\left(r\right) & < & \alpha_{i}{\left[1-v_{i}C_{i}\left(r\right)\right]}^{v_{i}/v_{0}}=\alpha_{i}{\left[1-v_{i}C_{i}\left(r\right)\right]}^{\gamma }{\left[1-v_{i}C_{i}\left(r\right)\right]}^{v_{i}/v_{0}-\gamma }\\ & < & \alpha_{i}\left[1-v_{i}C_{i}\left(r\right)\right]\left[1-\left(v_{i}/v_{0}-\gamma \right)v_{i}C_{i}\left(r\right)\right]\\ & < & \alpha_{i}\left[1-v_{i}C_{i}\left(r\right)\right]\;\mathrm{if}\;v_{i}/v_{0}>1, \end{array}$$

imply that the distributions are of Fermi-like type [87]

(7) $$\begin{array}{ccc} C_{i}\left(r\right) & < & \lim_{\alpha_{i}\to \infty }\frac{\alpha_{i}}{1+\alpha_{i}v_{i}^{2}/v_{0}}<\frac{v_{0}}{v_{i}^{2}}\;\mathrm{if}\;v_{i}/v_{0}\leq 1, \end{array}$$

(8) $$\begin{array}{ccc} C_{i}\left(r\right) & < & \lim_{\alpha_{i}\to \infty }\frac{\alpha_{i}}{1+\alpha_{i}v_{i}}<\frac{1}{v_{i}}\;\mathrm{if}\;v_{i}/v_{0}>1, \end{array}$$

i.e., $C_{i}\left(r\right)$ cannot exceed the maximum value $1/v_{i}^{2}$ or $1/v_{i}$ for any arbitrary (or even infinite) potential ϕ(r) at any location r in the domain $\Omega_{s}$, where i=1,⋯,K+1, $\alpha_{i}=C_{i}^{B}\exp\left(-\beta_{i}\phi \left(r\right)\right)/{\left(\Gamma^{B}\right)}^{v_{i}/v_{0}}>0$, $0<v_{i}/v_{0}-\gamma <1$, and γ≥1.

The classical Boltzmann distribution appears if all particles are treated as volumeless points, i.e., $v_{i}=0$ and $\Gamma \left(r\right)=\Gamma^{B}=1$. The classical Boltzmann distribution may produce an infinite concentration $C_{i}\left(r\right)\to \infty$ in crowded conditions when $-\beta_{i}\phi \left(r\right)\to \infty$, close to charged surfaces for example, which is physically impossible [64,65,66]. This is a major, even crippling deficiency of PB theory for modeling a system with strong local electric fields or interactions. The difficulty in the application of classical Boltzmann distributions to saturating systems has been avoided in the physiological literature (apparently starting with Hodgkin, Huxley, and Katz [88]) by redefining the Boltzmann distribution to deal with systems that can only exist in two states. This redefinition has been vital to physiological research and is used in hundreds of papers [89,90], but confusion results when the physiologists’ saturating two-state Boltzmann is not kept distinct from the unsaturating Boltzmann distribution of statistical mechanics [91].

It should be clearly understood that as beautiful as is Hodgkin’s derivation it begs the question of what physics creates and maintains two states. Indeed, it is not clear how one can define the word state in a usefully unique way in a protein of enormous molecular weight with motions covering the scale from femtoseconds to seconds.

The steric potential $S^{trc}\left(r\right)$ in Equation (4) first introduced in [64] is an entropic measure of crowding or emptiness of particles at r. If ϕ(r)=0 and $C_{i}\left(r\right)=C_{i}^{B}$ then $S^{trc}\left(r\right)=0$. The factor $v_{i}/v_{0}$ shows that the steric energy $\frac{-v_{i}}{v_{0}}S^{trc}\left(r\right)k_{B}T$ of a type *i* particle at r depends not only on the steric potential $S^{trc}\left(r\right)$ but also on its volume $v_{i}$ similar to the electric energy $\beta_{i}\phi \left(r\right)k_{B}T$ depending on both ϕ(r) and $q_{i}$ [72]. The steric potential $S^{trc}\left(r\right)$ is especially relevant to determining selectivity of specific ions by certain biological ion channels [65,66,68,70,72].

In this mean-field Fermi distribution, it is impossible for a volume $v_{i}$ to be completely filled with particles, i.e., it is impossible to have $v_{i}C_{i}\left(r\right)=1$ (and thus Γ(r)=0) since that would make $S^{trc}\left(r\right)=-\infty$ and hence $C_{i}\left(r\right)=0$, a contradiction. Therefore, *we must include the voids as a separate species if we treat ions and water having volumes in a model* for which $C_{i}\left(r\right)<1/v_{i}$ and Γ(r)≠0 for all i=1,⋯,K+1 and r∈$\Omega_{s}$. This is a critical property distinguishing our theory from others that do not consider water as a molecule with volume and so do not have to consider voids. We shall elaborate this property in Section 4.

Our theory is consistent with the classical theory of van der Waals in molecular physics, which describes nonbond interactions between any pair of atoms as a distance-dependent potential such as the Lennard-Jones (L-J) potential that cannot have zero distance between the pair [85,86]. Indeed, the steric potential $S^{trc}\left(r\right)$ can be written as a function of the volume of all molecular species (of course, including water as well as ions). Classical extensions of van der Waals theories often use this variable, but seem not to mention the existence or importance of voids.

The steric potential $S^{trc}\left(r\right)$ lumps all van der Waals potential energies of paired particles in a mean-field sense. It is an approximation of L-J potentials that describe local variations of L-J distances (and thus empty voids) between any pair of particles. L-J potentials are highly oscillatory and extremely expensive and unstable to compute numerically [66]. Calculations that involve L-J potentials [92,93,94,95,96,97,98], or even truncated versions of L-J potentials [99,100,101] must be extensively checked to be sure that results do not depend on irrelevant parameters. Any description that uses L-J potentials has a serious problem specifying the combining rule. The details of the combining rule *directly* change predictions of effects of different ions (selectivity) and so predictions depend on the reliability of data that determines the combining rule and its parameters.

*The steric potential does not require combining rules*. Since we consider specific sizes of ions and water with voids, the steric potential is valid on the *atomic scale* of L-J potentials. It is also *consistent* with that on the *macroscopic scale* of continuum models as shown in Section 6 and Section 8.

To our surprise during the writing of this article, we found Equation (2) in Bikerman’s 1942 paper [42] is *exactly* the *same* as Equation (4) for a special case of binary ionic liquids with the identical steric energies of different-sized ions, i.e., the factor $v_{i}/v_{0}=1$ in (4). The steric potential in Equation (4) is however not explicitly expressed in Bikerman’s paper. Therefore, Bikerman’s concentration function is a Fermi distribution, a generic term used in statistical mechanics. We do NOT use exactly the Fermi distribution as Fermi derived in 1926 for identical particles now called fermions in quantum mechanics. So it is both more precise and historically correct to use the name “Poisson–Bikerman” equation for finite-sized ions as a generalization of the Poisson–Boltzmann equation for volumeless ions in electrochemical and bioelectric systems.

As noted by Bazant et al. in their review paper [14], Bikerman’s paper has been poorly cited in the literature until recently. In our intensive and extensive study of the literature since 2013 [64], we have never found any paper specifically using Bikerman’s formula as Equation (4), although of course there may be an instance we have not found. We thus now change the term “Poisson-Fermi” used in our earlier papers to “Poisson-Bikerman” in honor of Bikerman’s brilliant work. We present here mathematical as well as physical justifications of a very general treatment of different-sized ions and water molecules in the mean-field framework based on Bikerman’s pioneer work.

## 3. Fourth-Order Poisson-Bikerman Equation and Correlations

Electrolytes have been treated mostly in the tradition of physical chemistry of isolated systems that proved so remarkably successful in understanding the properties of ideal gases in atomic detail, long before the theory of partial differential equations, let alone numerical computing was developed. Most applications of ionic solutions however involve systems that are not at all isolated. Rather, most practical systems include electrodes to deliver current and control potential, and reservoirs to manipulate the concentrations and types of ions in the solution. Indeed, all biology occurs in ionic solutions and nearly all of biology involves large flows. It is necessary then to extend classical approaches so they deal with external electric fields and other boundary conditions and allow flow so the theory can give useful results that are applicable to most actual systems.

When the electrolyte system in $\Omega_{s}$ is subject to external fields such as applied voltages, surface charges, and concentration gradients on the boundary $\partial \Omega_{s}$, the electric field E(r) of the system, the displacement field D(r) of free ions, and the polarization field P(r) of water are generated at all r in $\Omega_{s}$. In Maxwell’s theory [102,103], these fields form a constitutive relation

(9) $$D\left(r\right)=\epsilon_{0}E\left(r\right)+P\left(r\right)$$

and the displacement field satisfies

(10) $$\nabla \cdot D\left(r\right)=\rho_{ion}\left(r\right)=\sum_{i=1}^{K}q_{i}C_{i}\left(r\right),$$

where $\epsilon_{0}$ is the vacuum permittivity, $\rho_{ion}\left(r\right)$ is the charge density of ions, and $C_{i}\left(r\right)$ are the concentrations defined in (4). See [104] for a modern formulation of Maxwell’s theory applicable wherever the Bohm version of quantum mechanics applies [105,106].

The electric field E(r) is thus screened by water (Bjerrum screening) and ions (Debye screening) in a correlated manner that is usually characterized by a correlation length $l_{c}$ [77,78,107]. The screened force between two charges in ionic solutions (at r and $r^{\prime }$ in $\Omega_{s}$) has been studied extensively in classical field theory and is often described by the van der Waals potential kernel [71,72,84,107,108]

(11) $$W\left(r-r^{\prime }\right)=\frac{e^{-\left|r-r^{\prime }\right|/l_{c}}}{\left|r-r^{\prime }\right|/l_{c}}$$

that satisfies the Laplace-Poisson equation [108]

(12) $$-\Delta W\left(r-r^{\prime }\right)+\frac{1}{l_{c}^{2}}W\left(r-r^{\prime }\right)=\delta \left(r-r^{\prime }\right),\;\;r,\;r^{\prime }\in R^{3}$$

in the whole space $R^{3}$, where $\Delta =\nabla \cdot \nabla =\nabla^{2}$ is the Laplace operator with respect to r and $\delta (r-r^{\prime })$ is the Dirac delta function at $r^{\prime }$.

The potential $\tilde{\phi }\left(r\right)$ defined in

(13) $$D\left(r\right)=-\epsilon_{s}\nabla \tilde{\phi }\left(r\right)$$

describes an electric potential of free ions [72,107] that are correlated only by the mean electric field according to the Poisson equation

(14) $$-\epsilon_{s}\Delta \tilde{\phi }\left(r\right)=\rho_{ion}\left(r\right),$$

a second-order partial differential equation, where $\epsilon_{s}=\epsilon_{w}\epsilon_{0}$ and $\epsilon_{w}$ is the dielectric constant of water. This potential does not account for correlation energies between individual ions or between ion and polarized water in high field or crowded conditions under which the size and valence of ions and the polarization of water play significant roles [17,65,66,67,68,77,78,107].

The correlations implicit in Maxwell’s equations are of the mean-field and can be summarized by the statement that current is conserved perfectly and universally on all scales that the Maxwell equations are valid, where current includes the term $\epsilon_{0}\frac{\partial E(r,t)}{\partial t}$. This term allows the Maxwell equations to describe the propagation of light through a vacuum, and it allows charge to be relativistically invariant, i.e., independent of velocity unlike mass, length, and time all of which vary dramatically as velocities approach the speed of light [104,106].

We introduce the *correlated* electric potential

(15) $$\phi \left(r\right)=\int_{R^{3}}\frac{1}{l_{c}^{2}}W\left(r-r^{\prime }\right)\tilde{\phi }\left(r^{\prime }\right)dr^{\prime }$$

in [72] as a convolution of the displacement potential $\tilde{\phi }\left(r^{\prime }\right)$ with $W(r-r^{\prime })$ to deal with the correlation and polarization effects in electrolyte solutions. However, it would be too expensive to calculate ϕ(r) using (15). Multiplying (12) by $\tilde{\phi }\left(r^{\prime }\right)$ and then integrating over $R^{3}$ with respect to $r^{\prime }$ [71], we obtain

(16) $$-l_{c}^{2}\Delta \phi \left(r\right)+\phi \left(r\right)=\tilde{\phi }\left(r\right)$$

a Laplace-Poisson equation [107,108] that satisfies (15) in the whole unbounded space $R^{3}$ with the boundary conditions $\phi \left(r\right)=\tilde{\phi }\left(r\right)=0$ at infinity. From (14) and (16), we obtain the *4th-order* Poisson–Bikerman equation

(17) $$\epsilon_{s}\left[l_{c}^{2}\Delta -1\right]\Delta \phi \left(r\right)=\rho_{ion}\left(r\right),\;r\in \Omega_{s},$$

a PDE that is an approximation of (16) in a bounded domain $\Omega_{s}\subset$$R^{3}$ with suitable boundary conditions (see below) of ϕ(r) on $\partial \Omega_{s}$. We can thus use (9) to find the polarization field

(18) $$P\left(r\right)=\epsilon_{s}l_{c}^{2}\nabla \left(\Delta \phi \left(r\right)\right)-\left(\epsilon_{w}-1\right)\epsilon_{0}\nabla \phi \left(r\right)$$

with E(r)=−∇ϕ(r). If $l_{c}=0$, we recover the standard Poisson Equation (14) and the standard polarization $P=\epsilon_{0}\left(\epsilon_{w}-1\right)E$ with the electric susceptibility $\epsilon_{w}-1$ (and thus the dielectric constant $\epsilon_{w}$) if water is treated as a time independent, isotropic, and linear dielectric medium [103]. In this case, the field relation $D=\epsilon_{w}\epsilon_{0}E$ with the scalar constant permittivity $\epsilon_{s}\epsilon_{0}$ is an approximation of the exact relation (9) due to the simplification of the dielectric responses of the medium material to the electric field E [109,110,111].

The exponential van der Waals potential $W\left(r-r^{\prime }\right)=\frac{e^{-\left|r-r^{\prime }\right|/l_{c}}}{\left|r-r^{\prime }\right|/l_{c}}$ [84] is called the Yukawa [112] potential in [71,72] and usually in physics, which is an anachronism [108,113]. Van der Waals derived this potential in his theory of capillarity based on the proposition that the intermolecular potential of liquids and gases is shorter-ranged, but much stronger than Coulomb’s electric potential [108]. Ornstein and Zernike (OZ) introduced short- (direct) and long-ranged (indirect) correlation functions in their critical point theory [114]. There are three important properties of the van der Waals potential: (i) it satisfies the Laplace-Poisson Equation (12), (ii) it generates the same functional form for short- and long-ranged correlations in the OZ theory, and (iii) it solves van der Waals’s problem for the intermolecular potential [108].

Therefore, the potential ϕ(r) in (15) includes *correlation* energies of *ion-ion* and *ion-water* interactions in *short* as well as *long* ranges in our system. The *correlation length* $l_{c}$ can be derived from the OZ equation, see Equation (13) in [108], but the derivation is not very useful. The correlation length becomes an unknown functional of $\rho_{ion}\left(r\right)$ in (10) and the OZ direct correlation function, and is hence usually chosen as an empirical parameter to fit experimental, molecular dynamics (MD), or Monte Carlo (MC) data [14,64,65,66,67,68,69,70,71,72,73,75,77,78,107]. It seems clear that it would be useful to have a theory that showed the dependence of correlation length on ion composition and concentration, and other parameters.

There are several approaches to fourth-order Poisson-Boltzmann equations for modeling ion-ion and ion-water correlations from different perspectives of physics [71,77,78,115,116]. In [77], a decomposed kernel acts on a charge density of counterions in a binary liquid without volumes and water (ion-ion correlations) in contrast to the potential $\tilde{\phi }\left(r\right)$ in (15) that is generated by different-sized ions and water with voids in (14) (ion-ion and ion-water correlations in a multi-component aqueous electrolyte). The kernel consists of short-range (of van der Waals type) and long-range components from a decomposition of Coulomb’s interactions. In [78], the kernel is a general nonlocal kernel that acts on a charge density of equal-sized ions in a binary liquid without water (ion-ion correlations). The kernel is a series expansion of the gradient operator ∇ and thus can yield not only a fourth-order PB but even higher-order PDEs. The fourth-order PB is the first-order approximation of the energy expansion that converges only with small wavenumbers *k* in Fourier frequency domain for the dielectric response of ionic liquids [78].

Derived from the framework of nonlocal electrostatics for modeling the dielectric properties of water in [107], the kernel acting on $\tilde{\phi }\left(r\right)$ in [71] (ion-ion and ion-water correlations) consists of a van der Waals function and the Dirac delta function that correspond to the limiting cases k=0 and k=∞, respectively. In [115], a system of three PDEs derived from electrostatics and thermodynamic pressure has electric potential and concentration gradients of equal-sized cations and anions in a binary fluid as three unknown functions. Linearization and simplification of the nonlinear system can yield a linear fourth-order PB (ion-ion correlations). In [116], the fourth-order PB is derived from a free energy functional that models ion-ion correlations in a binary liquid using volume-fraction functions of equal-sized cations and anions with two additional parameters associated with the interaction energies of these two functions and their gradients.

The *dielectric operator* $\epsilon_{s}\left(l_{c}^{2}\Delta -1\right)$ in (17) describes changes in dielectric response of water with salt concentrations (ion-water correlations), ion-ion correlations, and water polarizations all via the mean-field charge density function $\rho_{ion}\left(r\right)$ provided that we can solve (4) and (17) for a consistent potential function ϕ(r). Therefore, the operator (a mapping) depends not only on ion and water concentrations ($C_{i}^{B}$ for all arbitrary species i=1,⋯,K+1 of particles with any arbitrary shapes and volumes) but also on the location r and the voids at r. The operator thus produces a *dielectric function* $\hat{\epsilon }\left(r,C_{i}^{B}\right)$ as an *output* from the solution ϕ(r) that satisfies the 4PBik (17) that saturates as a function of concentration (4), as we shall repeatedly emphasize. This dielectric function $\hat{\epsilon }\left(r,C_{i}^{B}\right)$ is not an additional model for $\tilde{\epsilon }\left(r\right)$, $\tilde{\epsilon }\left(k\right)$, or $\tilde{\epsilon }\left(C_{i}^{B}\right)$ as it often is in other models in the literature [62,63,117,118,119,120,121,122,123,124,125,126,127]. Here the dielectric function is an output, as we have stated.

The 4PBik Equation (17) with (4) is a very general model using only one extra parameter $l_{c}$ in the fourth-order operator to include many physical properties ignored by the classical Poisson-Boltzmann equation. We shall illustrate these properties of our model in Section 8.

## 4. Generalized Gibbs Free Energy Functional

To generalize the Gibbs free energy functional for Boltzmann distributions that satisfy the classical Poisson–Boltzmann equation [3,128,129], we introduce a functional in [72] for saturating Fermi distributions (4) that satisfy the 4^th^-order Poisson–Bikerman Equation (17)

(19) $$\begin{array}{cc} F(C) & =F_{el}\left(C\right)+F_{en}\left(C\right), \end{array}$$

(20) $$\begin{array}{cc} F_{el}\left(C\right) & =\frac{1}{2}\int_{\Omega_{s}}\rho_{ion}\left(r\right)L^{-1}\rho_{ion}\left(r\right)dr,\; \end{array}$$

(21) $$\begin{array}{cc} F_{en}\left(C\right) & =k_{B}T\int_{\Omega_{s}}\left\{\sum_{i=1}^{K+1}C_{i}\left(r\right)\left(\ln\frac{C_{i}\left(r\right)}{C_{i}^{B}}-1\right)+\frac{\Gamma (r)}{v_{0}}\left(\ln\frac{\Gamma (r)}{\Gamma^{B}}-1\right)\right\}dr, \end{array}$$

where $F_{el}\left(C\right)$ is an electrostatic functional, $F_{en}\left(C\right)$ is an entropy functional, $C=\left(C_{1}\left(r\right),\;C_{2}\left(r\right),\;\cdots ,\;C_{K+1}\left(r\right)\right)$, and $L^{-1}$ is the inverse of the self-adjoint positive linear operator $L=\epsilon_{s}\left(l_{c}^{2}\Delta -1\right)\Delta$ [71] in (17), i.e., $L\phi \left(r\right)=\rho_{ion}\left(r\right)$. C is a ‘concentration vector’ that specifies the number density, i.e., concentration of each species in the ionic solution, including water. C plays a central role in any theory of ionic solutions because it specifies the main property of a solution, namely its composition.

Taking the variations of F(C) at $C_{i}\left(r\right)$, we have

$$\begin{array}{c} \frac{\delta F(C)}{\delta C_{i}}=\int_{\Omega_{s}}\left\{k_{B}T\left[\ln\frac{C_{i}\left(r\right)}{C_{i}^{B}}-\frac{v_{i}}{v_{0}}\ln\frac{\Gamma (r)}{\Gamma^{B}}\right]+\frac{1}{2}\left(q_{i}L^{-1}\rho_{ion}\left(r\right)+\rho_{ion}\left(r\right)L^{-1}q_{i}\right)\right\}dr,\\ \frac{1}{2}\left(q_{i}L^{-1}\rho_{ion}\left(r\right)+\rho_{ion}\left(r\right)L^{-1}q_{i}\right)=q_{i}\phi \left(r\right), \end{array}$$

(22) $$\frac{\delta F(C)}{\delta C_{i}}=0\Rightarrow k_{B}T\left[\ln\frac{C_{i}\left(r\right)}{C_{i}^{B}}-\frac{v_{i}}{v_{0}}\ln\frac{\Gamma (r)}{\Gamma^{B}}\right]+q_{i}\phi \left(r\right)=0$$

that yields the saturating Fermi distributions in (4) for all i=1,⋯,K+1. Moreover, we have

(23) $$\frac{\delta^{2}F\left(C\right)}{\delta C_{i}^{2}}=\int_{\Omega_{s}}\left\{k_{B}T\left[\frac{1}{C_{i}\left(r\right)}+\frac{v_{i}^{2}}{v_{0}}\frac{\Gamma^{B}}{\Gamma (r)}\right]+q_{i}^{2}L^{-1}C_{i}\right\}dr>0$$

implying that the saturating Fermi distribution vector C is a unique minimizer of the functional F(C).

The Gibbs-Bikerman free energy functional F(C) has two important properties. First, its electrostatic part $F_{el}\left(C\right)$ is defined in terms of the composition vector C only. It depends only on concentrations and nothing else. If an electrostatic functional ${\tilde{F}}_{el}\left(\tilde{\phi }\left(r\right)\right)$ is defined in terms of ${\left|\nabla \tilde{\phi }\left(r\right)\right|}^{2}$ for the PB equation [64,78,124,130,131,132,133,134,135,136,137], the corresponding concentration vector $\tilde{C}$ and the potential $\tilde{\phi }\left(r\right)$ do *not* minimize the corresponding functional $\tilde{F}\left(\tilde{C},\tilde{\phi }\left(r\right)\right)$ [128,129], i.e., $\tilde{F}$ is not a Gibbs free energy functional [3,128]. Second, the limit of its entropic part

(24) $$\lim_{v_{i}\to 0}F_{en}\left(C\right)=k_{B}T\int_{\Omega_{s}}\sum_{i=1}^{K+1}C_{i}^{0}\left(r\right)\left(\ln\frac{C_{i}^{0}\left(r\right)}{C_{i}^{B}}-1\right)dr$$

exists ($F_{en}$ converges) when the volume $v_{i}$ tends to zero for all i=1,⋯,K+1. This implies that all ionic species have Boltzmann distributions $C_{i}^{0}\left(r\right)=C_{i}^{B}\exp\left(-\beta_{i}\phi \left(r\right)\right)$, i=1,⋯,K, the water concentration $C_{K+1}^{0}\left(r\right)=C_{K+1}^{B}$ is a constant, and the void fraction $\Gamma \left(r\right)=\Gamma^{B}=1$ since all particles are volumeless in PB theory. Therefore, the 4PBik model (4) and (17) is physically and mathematically *consistent* with the classical PB model in the limiting case when we ignore the steric ($v_{i}=0$) and correlation ($l_{c}=0$) effects.

There are many shortcomings of the lattice approach [138] frequently used to account for steric effects in lattice-based PB models [14,61,64,78,124,129,133,134,135,136,139,140,141]. For example, (i) it assumes equal-sized ions and thus cannot distinguish non-uniform particles as in (1), (ii) its effective ion size needs to be unrealistically large to fit data [14], (iii) its correction over Boltzmann’s point charge approach appears only at high surface charges [125], (iv) its pressure term diverges very weakly (is greatly underestimated) at close packing [142], and (v) its entropy functional may diverge as the volume of ions tends to zero, i.e., the corresponding *lattice-based PB* model is *not* physically and mathematically *consistent* with the classical PB model in the limiting case [66].

The importance of the restriction in Point (i) is hard to overstate. Almost all the interesting properties of ionic solutions arise because of their selectivity (as it is called in biology) or specificity between species, and those different properties arise in large measure because of the different diameters of the ions. The equal diameter case is dull and degenerate.

Point (v) is a critical problem that is closely related to Points (ii)–(iv). The divergence is obvious for an entropy term ${\tilde{F}}_{en}$ in Equation (2) in [133] as

(25) $$\lim_{v\to 0}{\tilde{F}}_{en}=\lim_{v\to 0}\sum_{i=1}^{K}{\tilde{C}}_{i}\left(r\right)\ln\left(v{\tilde{C}}_{i}\left(r\right)\right)=-\infty ,$$

which also appears in [61,64,78,124,129,133,134,135,136,139,140,141]. It is impossible to derive Boltzmann distributions ${\tilde{C}}_{i}\left(r\right)=C_{i}^{B}\exp\left(-\beta_{i}\tilde{\phi }\left(r\right)\right)$ from ${\tilde{F}}_{en}$ as v→0 without extra assumptions, see (2.6) in [129], for example. In fact, the assumption (2.6), i.e., $v{\tilde{C}}_{i}\left(r\right)>0$, actually forbids us from taking *v* to the limit zero.

Our derivation of $F_{en}\left(C\right)$ does not employ any lattice models but simply uses the exact volume Equation (1). Our theory should not be classified then as a lattice model as sometimes is the case, at least in informal discussions. The void function Γ(r) is an analytical generalization of the void fraction 1−Φ in (20) in [14] with all volume parameters $v_{i}$ (including the bulk fraction $\Gamma^{B}$) being physical instead of empirical as Φ. The excess chemical potential in [14] is $-k_{B}T\ln\left(1-\Phi \right)$ whereas ours is $F_{en}\left(C\right)$ in (21).

These expressions are different in important respects. Our model is not a lattice-based model because its differences are crucial both mathematically and physically. Indeed, the lattice-based model is in a certain sense internally inconsistent with classical statistical mechanics since a fundamental result of classical statistical mechanics $v{\tilde{C}}_{i}\left(r\right)>0$ prevents the model from satisfying the classical imperative of the Boltzmann distribution in the limit of zero *v*.

The Langmuir-type distribution

(26) $$C_{i}\left(x\right)=\frac{C_{i}^{B}\exp\left(-\beta_{i}\phi \left(x\right)\right)}{1+\sum_{j=1}^{K}\frac{C_{j}^{B}}{C_{j}^{\max}}\left(\exp\left(-\beta_{j}\phi \left(x\right)\right)-1\right)}\;$$

of different-sized ions (without water) proposed in [125] also reduces to a Boltzmann distribution as $v_{j}\to 0$, ∀j, where $C_{j}^{\max}=p/v_{j}$ and p≤1 is a packing parameter. This distribution saturates and thus is of Fermi type, i.e., $C_{i}\left(x\right)\leq C_{i}^{\max}$ and $v_{i}C_{i}\left(x\right)\leq 1$. The entropy term $-\ln\left(1+\sum_{j=1}^{K}\frac{C_{j}^{B}}{C_{j}^{\max}}\left(\exp\left(-\beta_{j}\phi \left(x\right)\right)-1\right)\right)$ does not involve voids so it is different from the $S^{trc}\left(r\right)$ in (4). Our distribution in (4) does not need any packing parameters and satisfies $v_{i}C_{i}\left(r\right)<1$.

## 5. Poisson-Nernst-Planck-Bikerman Model of Saturating Phenomena

For nonequilibrium systems, we can also generalize the classical Poisson-Nernst-Planck model [38,39,43,143,144] to the Poisson-Nernst-Planck–Bikerman model by coupling the flux density equation

(27) $$\frac{\partial C_{i}\left(r,t\right)}{\partial t}=-\nabla \cdot J_{i}\left(r,t\right),\;r\in \Omega_{s}$$

of each particle species i=1,⋯,K+1 (including water) to the 4PBik Equation (17), where the flux density is defined as

(28) $$J_{i}\left(r,t\right)=-D_{i}\left[\nabla C_{i}\left(r,t\right)+\beta_{i}C_{i}\left(r,t\right)\nabla \phi \left(r,t\right)-\frac{v_{i}}{v_{0}}C_{i}\left(r,t\right)\nabla S^{trc}\left(r,t\right)\right],$$

$D_{i}$ is the diffusion coefficient, and the time variable *t* is added to describe the dynamics of electric ϕ(r,t) and steric $S^{trc}\left(r,t\right)$ potentials.

The flux Equation (27) is called the Nernst-Planck-Bikerman equation because the steric potential $S^{trc}\left(r,t\right)$ is introduced into the classical NP equation so it can deal with saturating phenomena including those that arise from the unequal volumes of ions and the finite volume of molecular water. The PNPB model can be extended to include hydrodynamic kinetic and potential energies in the variational treatment of energy processes (i.e., EnVarA) by Hamilton’s least action and Rayleigh’s dissipation principles [145,146]. We shall however consider this as a topic for future work.

At equilibrium, the net flow of each particle species is a zero vector, i.e., $J_{i}\left(r\right)=0$ (in a steady state), which implies that

(29) $$\begin{array}{ccc} \nabla C_{i}\left(r\right)+\beta_{i}C_{i}\left(r\right)\nabla \phi \left(r\right)-\frac{v_{i}}{v_{0}}C_{i}\left(r\right)\nabla S^{trc}\left(r\right) & = & 0,\\ \nabla \left[C_{i}\left(r\right)\exp\left(\beta_{i}\phi \left(r\right)-\frac{v_{i}}{v_{0}}S^{trc}\left(r\right)\right)\right] & = & 0,\\ C_{i}\left(r\right)\exp\left(\beta_{i}\phi \left(r\right)-\frac{v_{i}}{v_{0}}S^{trc}\left(r\right)\right) & = & c_{i}, \end{array}$$

where the constant $c_{i}=C_{i}^{B}$ for $\phi \left(r\right)=S^{trc}\left(r\right)=0$. Therefore, (29) = (4), i.e., the NPB Equation (27) reduces to the saturating Fermi distribution (4) as the classical NP equation reduces to the Boltzmann distribution at equilibrium.

The gradient of the steric potential $\nabla S^{trc}\left(r,t\right)$ in (28) represents an entropic force of vacancies exerted on particles. The negative sign in $-C_{i}\left(r,t\right)\nabla S^{trc}\left(r,t\right)$ means that the steric force $\nabla S^{trc}\left(r,t\right)$ is in the opposite direction to the diffusion force $\nabla C_{i}\left(r,t\right)$.

Larger $S^{trc}\left(r,t\right)=\ln\frac{\Gamma (r,t)}{\Gamma^{B}}$ implies lower pressure because the ions occupy more space (less crowded) as implied by the numerator Γ(r,t). The larger the $S^{trc}\left(r,t\right)$ the lower pressure at the location r, the more the entropic force (the higher pressure) pushes particles to r from neighboring locations. The steric force is the opposite of the diffusion force $\nabla C_{i}\left(r,t\right)$ that pushes particles away from r if the concentration at r is larger than that at neighboring locations.

Moreover, the Nernst-Einstein relationship between diffusion and mobility [9] implies that the steric flux $D_{i}\frac{v_{i}}{v_{0}}C_{i}\left(r,t\right)\nabla S^{trc}\left(r,t\right)$ is greater if the particle is more mobile. The Nernst-Einstein relationship is generalized to

(30) $$\mu_{i}=v_{i}q_{i}D_{i}/\left(v_{0}k_{B}T\right),$$

where the mobility coefficient $\mu_{i}$ of an ion depends on its size $v_{i}$ in addition to its charge $q_{i}$. The mobility coefficient of water is $\mu_{K+1}=v_{K+1}D_{K+1}/\left(v_{0}k_{B}T\right)$. The drift term in (28) is thus $-D_{i}\beta_{i}C_{i}\left(r,t\right)\nabla \phi \left(r,t\right)=-\mu_{i}\left(v_{0}/v_{i}\right)C_{i}\left(r,t\right)\nabla \phi \left(r,t\right)$.

Therefore, the gradients of electric and steric potentials (∇ϕ(r,t) and $\nabla S^{trc}\left(r,t\right)$) describe the *charge/space competition* mechanism of particles in a crowded region within a mean-field framework. Since $S^{trc}\left(r,t\right)$ describes the dynamics of void movements, the dynamic crowdedness (pressure) of the flow system can also be quantified. A large amount of experimental data exists concerning the dependence of diffusion coefficient on the concentration and size of solutes. Comparing our model with this data is an important topic of future work.

The motion of water molecules, i.e., the *osmosis* of water [147,148] is directly controlled by the steric potential in our model and their distributions are expressed by $C_{K+1}\left(r,t\right)=C_{K+1}^{B}\exp\left(v_{K+1}S^{trc}\left(r,t\right)/v_{0}\right)$. Nevertheless, this motion is still implicitly changed by the electric potential ϕ(r,t) via the correlated motion of ions described by other $C_{j}\left(r,t\right)$ in the void fraction function Γ(r,t) and hence in the charge density $\rho_{ion}\left(r,t\right)$ in (17).

In summary, the PNPB model accounts for (i) the *steric* (*pressure*) effect of ions and water molecules, (ii) the *correlation* effect of crowded ions, (iii) the *screening* (*polarization*) effect of polar water, and (iv) the *charge*/*space competition* effect of ions and water molecules of different sizes and valences. These effects are all closely related to the interstitial voids between particles and described by two additional terms, namely the *correlation length* and the *steric potential*. The steric potential is most naturally written as a function of the volume of voids, but it can also be written as a function of the total volume of all molecules, including water and ions.

## 6. Generalized Debye-Hückel Theory

Thermodynamic modeling is of fundamental importance in the study of chemical and biological systems [1,6,9,11,12,13,16,32]. Since Debye and Hückel (DH) proposed their theory in 1923 [149] and Hückel extended it to include Born energy effects in 1925 [150], a great variety of extended DH models (equations of state) have been developed for modeling aqueous or mixed-solvent solutions over wide ranges of composition, temperature, and pressure [6,19,151,152,153,154,155]. Despite these intense efforts, robust thermodynamic modeling of electrolyte solutions still presents a difficult challenge for extended DH models due to an enormous number of parameters that need to be adjusted carefully and often subjectively [19,152,153,154,156].

It is indeed a frustrating despair (the word *frustration* on p. 11 in [16] and the word *despair* on p. 301 in [1]) that about *22,000* parameters [19] need to be extracted from the available experimental data for one temperature for combinatorial solutions of the most important 28 cations and 16 anions in salt chemistry by the Pitzer model [6], which is the most widely used DH model with unmatched precision for modeling electrolyte solutions [153]. The JESS (joint expert speciation system) is the world’s largest system of thermodynamic information relating to electrolytes, reactions in aqueous media, and hydrocarbon phase equilibria [157]. The total number of Pitzer’s fitting parameters in JESS is *95* [158].

By contrast, we propose in [75,76] a generalized Debye-Hückel theory from the 4PBik Equation (17) to include (i) steric effects, (ii) correlation effects, (iii) Born solvation energy, and (iv) ion hydration [159,160,161,162,163,164,165,166] that are missing in the original DH theory. The generalized theory can be used to calculate ion activities in all types of binary and multi-component solutions over wide ranges of concentration, temperature, and pressure with only *3* fitting parameters [69,73,75,76].

**Figure 1:**
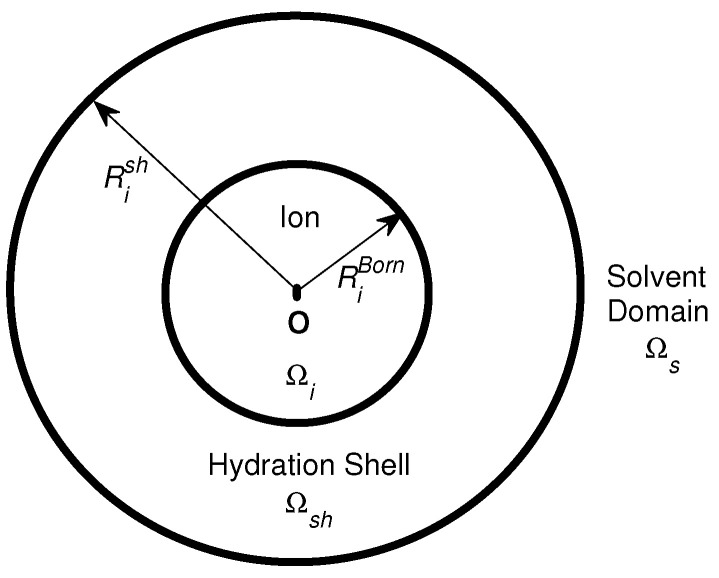
The model domain Ω is partitioned into the ion domain $\Omega_{i}$ (with radius $R_{i}^{Born}$), the hydration shell domain $\Omega_{sh}$ (with radius $R_{i}^{sh}$), and the remaining solvent domain $\Omega_{s}$.

We briefly outline the derivation of a generalized DH equation of state and refer to [76] for more details. The activity coefficient $\gamma_{i}$ of an ion of species *i* in an aqueous electrolyte solution with a total of *K* species of ions describes deviation of the chemical potential of the ion from ideality ($\gamma_{i}=1$) [11]. The excess chemical potential $\mu_{i}^{ex}=k_{B}T\ln\gamma_{i}$ can be calculated by [69,167]

(31) $$\mu_{i}^{ex}=\frac{1}{2}q_{i}\phi \left(0\right)-\frac{1}{2}q_{i}\phi^{0}\left(0\right),$$

where $q_{i}$ is the charge of the hydrated ion (also denoted by *i*), ϕ(r) is a reaction potential [167] function of spatial variable r in the domain $\bar{\Omega }$=${\bar{\Omega }}_{i}\cup {\bar{\Omega }}_{sh}\cup {\bar{\Omega }}_{s}$ shown in Figure 1, $\Omega_{i}$ is the spherical domain occupied by the ion *i*, $\Omega_{sh}$ is the hydration shell domain of the ion, $\Omega_{s}$ is the rest of solvent domain, 0 denotes the center (set to the origin) of the ion, and $\phi^{0}\left(r\right)$ is a potential function when the solvent domain $\Omega_{s}$ does not contain any ions at all with pure water only, i.e., when the solution is ideal. The radii of $\Omega_{i}$ and the outer boundary of $\Omega_{sh}$ are denoted by $R_{i}^{Born}$ (ionic cavity radius [160]) and $R_{i}^{sh}$, respectively.

The potential function ϕ(r) can be found by solving the 4PBik Equation (17) and the Laplace equation [69,73]

(32) $$\Delta \phi \left(r\right)=0\;\mathrm{in}\;\Omega_{i}\cup \Omega_{sh},$$

where $\epsilon_{s}$ is defined in ${\bar{\Omega }}_{sh}\cup \Omega_{s}$, the correlation length $l_{c}=\sqrt{l_{B}l_{D}/48}$ is a density-density correlation length independent of specific ionic radius [168], $l_{B}$ and $l_{D}$ are the Bjerrum and Debye lengths, respectively, the concentration $C_{k}\left(r\right)$ function (4) is defined in $\bar{\Omega }$ for all k=1,⋯,K+1 in molarity (M), and $v_{k}=4\pi a_{k}^{3}/3$ with radius $a_{k}$. Since the steric potential takes particle volumes and voids into account, the shell volume $V_{sh}$ of the shell domain $\Omega_{sh}$ can be determined by the steric potential $S_{sh}^{trc}=\frac{v_{0}}{v_{w}}\ln\frac{O_{i}^{w}}{V_{sh}C_{K+1}^{B}}=\ln\frac{V_{sh}-v_{w}O_{i}^{w}}{V_{sh}\Gamma^{B}}$ [69,73], where the occupant (coordination) number $O_{i}^{w}$ of water molecules is given by experimental data [166]. The shell radius $R_{i}^{sh}$ is thus determined and depends not only on $O_{i}^{w}$ but also on the bulk void fraction $\Gamma^{B}$, namely *on all salt and water bulk concentrations* ($C_{k}^{B}$) [69,73].

We reduce the complexity of higher-order approximations, and make them easier to implement by transforming the fourth-order PDE (17) to the following two second-order PDEs [64]

(33) $$\begin{array}{cc} \left(l_{c}^{2}\Delta -1\right)\psi \left(r\right) & =\rho_{ion}\left(r\right)\;\mathrm{in}\;\Omega_{s}, \end{array}$$

(34) $$\begin{array}{cc} \epsilon_{s}\Delta \phi \left(r\right) & =\psi \left(r\right)\;\mathrm{in}\;\Omega_{s}, \end{array}$$

where the extra unknown function ψ(r) is a density-like function as seen from (33) by setting $l_{c}=0$. The boundary and interface conditions for ϕ(r) and ψ(r) in (32)–(34) are [64]

(35) $$\begin{array}{cc} \phi (r) & =\psi \left(r\right)=0\;\mathrm{on}\;\partial \Omega_{s}\backslash \partial \Omega_{sh}, \end{array}$$

(36) $$\begin{array}{cc} \psi (r) & =-\rho_{s}\left(r\right)\;\mathrm{on}\;\partial \Omega_{sh}\cap \partial \Omega_{s}, \end{array}$$

(37) $$\begin{array}{cc} \left[\phi (r)\right] & =0\;\mathrm{on}\;\partial \Omega_{i}\cup \left(\partial \Omega_{sh}\cap \partial \Omega_{s}\right), \end{array}$$

(38) $$\begin{array}{cc} \left[\nabla \phi (r)\cdot n\right] & =0\;\mathrm{on}\;\partial \Omega_{sh}\cap \partial \Omega_{s}, \end{array}$$

(39) $$\begin{array}{cc} \left[\epsilon (r)\nabla \phi (r)\cdot n\right]\; & =\epsilon_{i}\nabla \phi^{*}\left(r\right)\cdot n\;\mathrm{on}\;\partial \Omega_{i}, \end{array}$$

where *∂* denotes the boundary of a domain, the jump function $\left[\phi \left(r\right)\right]=\lim_{r_{sh}\to r}\phi \left(r_{sh}\right)-\lim_{r_{i}\to r}\phi \left(r_{i}\right)$ at r $\in \partial \Omega_{i}$ with $r_{sh}\in$ $\Omega_{sh}$ and $r_{i}\in$ $\Omega_{i}$, $\epsilon \left(r\right)=\epsilon_{s}$ in $\Omega_{sh}$ and $\epsilon \left(r\right)=\epsilon_{ion}\epsilon_{0}$ in $\Omega_{i}$, $\epsilon_{ion}$ is a dielectric constant in $\Omega_{i}$, n is an outward normal unit vector at r∈ $\partial \Omega_{i}$, and $\phi^{*}\left(r\right)=q_{i}/\left(4\pi \epsilon_{i}\left|r-0\right|\right)$. Equation (32) avoids large errors in a direct approximation of the delta function δ(r−0) in the singular charge $q_{i}\delta \left(r-0\right)$ of the solvated ion at the origin 0 by transforming the singular charge to the Green’s function $\phi^{*}\left(r\right)$ on $\partial \Omega_{i}$ in (39) as an approximation source of the electric field produced by the solvated ion [169,170].

For simplicity, we consider a general binary (K=2) electrolyte C${}_{z_{2}}$A${}_{z_{1}}$ with the valences of the cation C${}^{z_{1}+}$ and anion A${}^{z_{2}-}$ being $z_{1}$ and $z_{2}$, respectively. The first-order Taylor approximation of the charge density functional $\rho_{ion}\left(\phi \left(r\right)\right)$ in (17) with respect to the electric potential ϕ(r) yields

(40) $$\rho_{ion}\left(\phi \left(r\right)\right)\approx \frac{-C_{1}^{B}q_{1}}{k_{B}T}\left[\left(q_{1}-q_{2}\right)-\Lambda q_{1}\right]\phi \left(r\right),$$

where $\Lambda =C_{1}^{B}{\left(v_{1}-v_{2}\right)}^{2}/\left[\Gamma^{B}v_{0}+\left(v_{1}^{2}C_{1}^{B}+v_{2}^{2}C_{2}^{B}+v_{3}^{2}C_{3}^{B}\right)\right]$ which is a quantity corresponding to a linearization of the steric potential $S^{trc}\left(r\right)$ [76]. Consequently, we obtain a *generalized Debye length*

(41) $$l_{D4PBik}={\left(\frac{\epsilon_{s}k_{B}T}{C_{1}^{B}\left(\left(1-\Lambda \right)q_{1}^{2}-q_{1}q_{2}\right)}\right)}^{1/2}$$

that reduces to the original Debye length $l_{D}$ [11] if $v_{1}=v_{2}\neq 0$ (two ionic species with equal radius and thus Λ=0) or $v_{1}=v_{2}=$ $v_{3}=0$ (all particles treated as volumeless points in standard texts for PB [11]). The nonlinear value of Λ≠0 for $v_{1}=v_{2}\neq 0$ can be obtained by Newton’s method [76].

Equation (33) is a second-order PDE that requires two boundary conditions like (35) and (36) for a unique solution ψ(r). Since ψ(r)= $\epsilon_{s}\nabla^{2}\phi \left(r\right)=-\rho \left(r\right)\approx \epsilon_{s}\kappa^{2}\phi \left(r\right)$ if $l_{c}=0$, Equation (36) is a simplified (approximate) boundary condition for ψ(r) on $\partial \Omega_{sh}\cap \partial \Omega_{s}$ without involving higher-order derivatives of ψ(r) (or the third-order derivative of ϕ(r)). The approximations in (36) and (40) do not significantly affect our generalized DH model’s ability to fit activity data. However, these assumptions should be carefully scrutinized in other applications such as highly charged surfaces. Bazant et al. have recently developed more consistent and general boundary conditions for their fourth-order model by enforcing continuity of the Maxwell stress at a charged interface [171,172].

In [76], we analytically solve the linear 4PBik PDEs (32), (33), and (34) with (40) in a similar way as Debye and Hückel solved the linear PB equation for a spherically symmetric system. However, the spherical domain shown in Figure 1 and the boundary and interface conditions in (35)–(39) are different from those of the standard method for the linear PB equation in physical chemistry texts [11]. The analysis consists of the following steps: (i) The nonlinear term $\rho_{ion}\left(r\right)$ in (33) is linearized to the linear term $-\epsilon_{s}\phi /l_{D4PBik}^{2}$ in (40) as that of Debye and Hückel. (ii) The linear PDEs corresponding to (33) and (34) are then formulated into a system of eigenvalue problems with eigenfunctions $\left(\phi (r),\;\psi (r)\right)$ and eigenvalues $\left(\lambda_{1},\;\lambda_{2}\right)$, where the general solution of ϕ(r) is equal to that of Debye and Hückel in the solvent domain $\Omega_{s}$ (not the entire domain) when $l_{c}=v_{1}=v_{2}=$$v_{3}=0$. (iii) A unique pair of eigenfunctions $\left(\phi^{4PBik}\left(r\right),\;\psi^{4PBik}\left(r\right)\right)$ is found under conditions (35)–(39), where $\phi^{4PBik}\left(r\right)$ is equal to that of Debye and Hückel in $\Omega_{s}$ when $l_{c}=v_{1}=v_{2}=$$v_{3}=0$.

The analytical potential function that we found [76] is

(42) $$\phi^{4PBik}\left(r\right)=\left\{\begin{array}{c} \frac{q_{i}}{4\pi \epsilon_{s}R_{i}^{Born}}+\frac{q_{i}}{4\pi \epsilon_{s}R_{i}^{sh}}\left(\Theta -1\right)\;\mathrm{in}\;\Omega_{i}\\ \frac{q_{i}}{4\pi \epsilon_{s}r}+\frac{q_{i}}{4\pi \epsilon_{s}R_{i}^{sh}}\left(\Theta -1\right)\;\mathrm{in}\;\Omega_{sh}\\ \frac{q_{i}}{4\pi \epsilon_{s}r}\left[\frac{\lambda_{1}^{2}e^{-\sqrt{\lambda_{2}}\left(r-R_{i}^{sh}\right)}-\lambda_{2}^{2}e^{-\sqrt{\lambda_{1}}\left(r-R_{i}^{sh}\right)}}{\lambda_{1}^{2}\left(\sqrt{\lambda_{2}}R_{i}^{sh}+1\right)-\lambda_{2}^{2}\left(\sqrt{\lambda_{1}}R_{i}^{sh}+1\right)}\right]\;\mathrm{in}\;\Omega_{s}, \end{array}\right.$$

where

(43) $$\Theta =\frac{\lambda_{1}^{2}-\lambda_{2}^{2}}{\lambda_{1}^{2}\left(\sqrt{\lambda_{2}}R_{i}^{sh}+1\right)-\lambda_{2}^{2}\left(\sqrt{\lambda_{1}}R_{i}^{sh}+1\right)},$$

$r=\left|r\right|$, $\lambda_{1}=\left(1-\sqrt{1-4l_{c}^{2}/l_{D4PBik}^{2}}\right)/\left(2l_{c}^{2}\right)$, and $\lambda_{2}=\left(1+\sqrt{1-4l_{c}^{2}/l_{D4PBik}^{2}}\right)/\left(2l_{c}^{2}\right)$. Please note that $\lim_{l_{c}\to 0}\lambda_{1}=1/l_{D4PBik}^{2}$, $\lim_{l_{c}\to 0}\lambda_{2}=\infty$, and $\lim_{l_{c}\to 0}\Theta =\lim_{C_{1}^{B}\to 0}\Theta =\lim_{l_{D4PBik}\to \infty }\Theta =1$ [76]. The linearized 4PBik potential $\phi^{4PBik}\left(r\right)$ reduces to the linearized PB potential $\phi^{PB}\left(r\right)=q_{i}e^{-r/l_{D}}/\left(4\pi \epsilon_{s}r\right)$ as in standard texts (e.g., Equation (7.46) in [11]) by taking $\lim_{l_{c}\to 0}\phi^{4PBik}\left(r\right)$ with $v_{k}=0$ for all *k*, $R_{i}^{sh}=0$, and r>0 [76].

As discussed in [173], since the solvation free energy of an ion *i* varies with salt concentrations, the Born energy $q_{i}^{2}\left(\frac{1}{\epsilon_{w}}-1\right)/8\pi \epsilon_{0}R_{i}^{0}$ in pure water (i.e., $C_{i}^{B}=0$) with a constant Born radius $R_{i}^{0}$ should change to depend on $C_{i}^{B}\geq 0$. Equivalently, the Born radius $R_{i}^{Born}$ in (42) is variable and we can model it from $R_{i}^{0}$ by a simple formula [69,73]

(44) $$R_{i}^{Born}=\theta R_{i}^{0},\;\;\;\theta =1+\alpha_{1}^{i}{\left({\bar{C}}_{i}^{B}\right)}^{1/2}+\alpha_{2}^{i}{\bar{C}}_{i}^{B}+\alpha_{3}^{i}{\left({\bar{C}}_{i}^{B}\right)}^{3/2},$$

where ${\bar{C}}_{i}^{B}=$$C_{i}^{B}$/M is a dimensionless bulk concentration and $\alpha_{1}^{i}$, $\alpha_{2}^{i}$, and $\alpha_{3}^{i}$ are parameters for modifying the experimental Born radius $R_{i}^{0}$ to fit experimental activity coefficient $\gamma_{i}$ that changes with the bulk concentration $C_{i}^{B}$ of the ion. The Born radii $R_{i}^{0}$ given below are from [173] obtained from the experimental hydration Helmholtz free energies of those ions given in [12]. The three parameters in (44) have physical or mathematical meanings unlike many parameters in the Pitzer model [19,153,156]. The first parameter $\alpha_{1}^{i}$ adjusts $R_{i}^{0}$ and accounts for the real thickness of the ionic atmosphere (Debye length), which is proportional to the square root of the ionic strength in the DH theory [11]. The second $\alpha_{2}^{i}$ and third $\alpha_{3}^{i}$ parameters are adjustments in the next orders of approximation beyond the DH treatment of ionic atmosphere [73].

The potential value $\phi^{0}\left(0\right)=\lim_{C_{1}^{B}\to 0}\phi^{4PBik}\left(0\right)=$$q_{i}/\left(4\pi \epsilon_{s}R_{i}^{0}\right)$ by $\lim_{C_{1}^{B}\to 0}\Theta =1$ and $\lim_{C_{1}^{B}\to 0}R_{i}^{Born}=R_{i}^{0}$. From (31) and (42), we thus have a generalized activity coefficient $\gamma_{i}^{4PBik}$ in

(45) $$\ln\gamma_{i}^{4PBik}=\frac{q_{i}^{2}}{8\pi \epsilon_{s}k_{B}T}\left(\frac{1}{R_{i}^{Born}}-\frac{1}{R_{i}^{0}}+\frac{\Theta -1}{R_{i}^{sh}}\right),$$

which satisfies the DH limiting law, i.e., $\gamma_{i}^{4PBik}=\gamma_{i}^{DH}=1$ for infinite dilute (ideal) solutions as $C_{i}^{B}\to 0$. The generalized activity coefficient $\gamma_{i}^{4PBik}$ reduces to the classical $\gamma_{i}^{DH}$ proposed by Debye and Hückel in 1923 [149], namely

(46) $$\ln\gamma_{i}^{DH}=\frac{-q_{i}^{2}}{8\pi \epsilon_{s}k_{B}Tl_{D}\left(1+R_{i}^{sh}/l_{D}\right)}$$

if $R_{i}^{Born}=R_{i}^{0}$ (without considering Born energy effects), $R_{i}^{sh}=R_{i}$ (an effective ionic radius [149]), $l_{D4PBik}=l_{D}$ (no steric effect), and $l_{c}=0$ (no correlation effect). The reduction shown in [76] is by taking the limit of the last term in (45) as $l_{c}\to 0$, i.e., $\lim_{l_{c}\to 0}\frac{\Theta -1}{R_{i}^{sh}}=\frac{-1}{R_{i}+l_{D}}.$

Hückel soon realized that the DH formula (46) failed to fit experimental data at high ionic strengths and modified it in 1925 [150] by adding one more parameter $\eta_{1}$ to become (see Equation (7.115) in [11])

(47) $$\ln\gamma_{i}^{DHB}=\frac{-q_{i}^{2}}{8\pi \epsilon_{s}k_{B}Tl_{D}\left(1+\eta_{0}\sqrt{I}\right)}+\eta_{1}I,$$

where $\eta_{0}$ (an approximation of $R_{i}^{sh}$) and $\eta_{1}$ account for the distance of closest approach to the ion *i* and the salting-out effect (an approximation of the Born energy), respectively [11], where $I=\frac{1}{2}\sum_{i}C_{i}^{B}z_{i}^{2}$ is the ionic strength of the solution. Consequently, a variety of extended DH models $\gamma_{i}^{DHBx}$ [153,174] in the form similar to

(48) $$\ln\gamma_{i}^{DHBx}=\frac{-q_{i}^{2}}{8\pi \epsilon_{s}k_{B}Tl_{D}\left(1+\eta_{0}\sqrt{I}\right)}+\sum_{k\neq 0}\eta_{k}I^{k}$$

have been proposed in the literature to express other thermodynamic properties such as temperature and pressure by a power expansion of *I* with more and more parameters $\eta_{k}$ that can increase combinatorially with various composition, temperature, and pressure to a frustrating amount [1,16,19]. Please note that $\eta_{k}$ may also depend on ionic strength *I* in a complicated way, see e.g., Equation (2) in [153]. Many expressions of those parameters are rather long and tedious and do not have clear physical meaning [19,153,156]. The Davies equation [175] is a special form of (47) with a linear term in *I*.

The $R_{i}^{Born}$ term in (45) *differs significantly* from the last term in (48) as they are the *inverse* of each other in terms of *I* and parameters, i.e., *I*, $\alpha_{1}$, $\alpha_{2}$, and $\alpha_{3}$ are in the denominator in (45) whereas *I* and $\eta_{k}$ are in the numerator in (48). This implies that $\gamma_{i}^{4PBik}$ and $\gamma_{i}^{DHBx}$ vary oppositely with *I*. Consequently, the values of $\alpha_{1}$, $\alpha_{2}$, and $\alpha_{3}$ are totally different from those of $\eta_{k}$ when we use $\gamma_{i}^{4PBik}$ and $\gamma_{i}^{DHBx}$ to fit experimental activity coefficients with *I* varying from low to high values [76]. This may explain why the empirical nature of extended DH models requires a great deal of effort to extract parameters (without physical hints) from existent thermodynamic databases by regression analysis [19,153].

## 7. Numerical Methods

Numerical simulations are indispensable to study chemical, physical, and mathematical properties of biological and chemical systems in realistic applications, especially with experimental details at atomic scale such as ion channels in the Protein Data Bank (PDB) [57]. Continuum PDE models have substantial advantages over Monte Carlo, Brownian dynamics (BD), or molecular dynamics in physical insights and computational efficiency that are of great importance in studying a range of conditions and concentrations especially for large nonequilibrium or inhomogeneous systems, as are present in experiments and in life itself [10,17,21,95,121,176,177,178,179,180,181,182,183,184,185].

The literature on numerical methods for solving PB and PNP models is vast [64,68,74]. We summarize here some important features of the methods proposed in [64,68,74] for Poisson-Bikerman and Poisson-Nernst-Planck-Bikerman models, which may be useful for workers in numerical analysis and coding practice. Since PNPB including 4PBik is highly nonlinear and the geometry of protein structures is very complex, we emphasize two different types of methods, namely nonlinear iterative methods and discretization methods for these two problems as follows.

### 7.1. Nonlinear Iterative Methods

**Figure 2:**
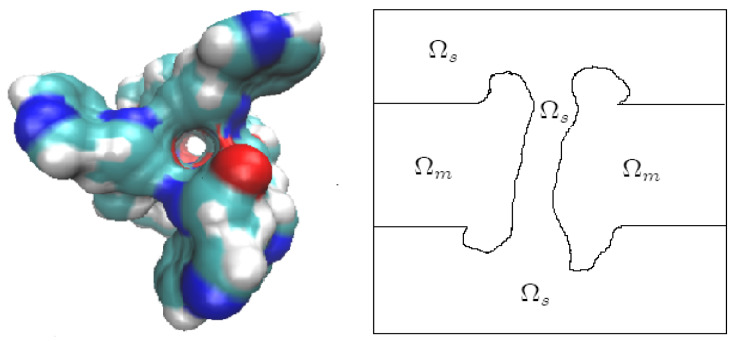
**Left (L)**: Top view of the gramicidin A channel. **Right (R)**: A cross section of 3D simulation domain for the channel placed in a rectangular box, where $\Omega_{m}$ is the biomolecular domain consisting of the channel protein and the membrane and $\Omega_{s}$ is the solvent domain consisting of the channel pore and the baths.

For the PNPB system of K+1 NP equations in (27), Laplace Equation (32), and two 4PBik equations in (33) and (34), the total number of second-order PDEs that we need to solve is K+4. These PDEs are coupled together and highly nonlinear except (32). Numerically solving this kind of nonlinear systems is not straightforward [64,68,74]. We use the following algorithm to explain essential procedures for solving the steady-state PNPB system, where $\Omega_{m}$ denotes the biomolecular domain that contains a total of *Q* fixed atomic charges $q_{j}$ located at $r_{j}$ in a channel protein as shown in Figure 2L for the gramicidin A channel downloaded from PDB with Q=554, for example, $\partial \Omega_{m}$ denotes the molecular surface of the protein and the membrane lipids through which the protein crosses as shown in Figure 2R, and $\Omega_{s}$ is the solvent domain consisting of the channel pore and the extracellular and intracellular baths for mobile ions and water molecules.

*Nonlinear Iterative Algorithm [68]:*

1. Solve Laplace Equation $-\nabla^{2}\phi \left(r\right)=0$ for $\phi^{0}\left(r\right)$ in $\Omega_{m}$ once for all with $\phi^{0}\left(r\right)=\phi^{*}\left(r\right)=\sum_{j=1}^{Q}q_{j}/\left(4\pi \epsilon_{m}\epsilon_{0}\left|r-r_{j}\right|\right)$ on $\partial \Omega_{m}$.
2. Solve Poisson Equation $-\nabla \cdot \left(\epsilon \nabla \phi (r)\right)=\rho_{ion}\left(r\right)$ for $\phi^{Old}\left(r\right)$ in $\Omega_{s}$ with $\rho_{ion}\left(r\right)=0$, $\phi^{Old}=V=0$ on ∂Ω, and the jump condition $\left[\epsilon \nabla \phi^{Old}\cdot n\right]=-\epsilon_{m}\epsilon_{0}\nabla \left(\phi^{*}+\phi^{0}\right)\cdot n$ on $\partial \Omega_{m}$ as (39), where *V* denotes applied voltage.
3. $V=V_{0}\neq 0$ an initial voltage.
4. Solve 4PBik1 Equation $\epsilon_{s}\left(\lambda_{c}l_{c}^{2}\nabla^{2}-1\right)\Psi \left(r\right)=\sum_{i=1}^{K}q_{i}C_{i}^{Old}\left(r\right)$ for $\Psi^{New}\left(r\right)$ in $\Omega_{s}$ with $\nabla \Psi^{New}\cdot n=0$ on $\partial \Omega_{m}$, $\Psi^{New}=0$ on ∂Ω, $C_{i}^{Old}\left(r\right)=C_{i}^{B}\exp\left(-\beta_{i}\phi^{Old}\left(r\right)+\frac{v_{i}}{v_{0}}S^{trc}\left(r\right)\right)$, $S^{trc}\left(r\right)=\ln\frac{\Gamma^{Old}\left(r\right)}{\Gamma^{B}}$, and $\Gamma^{Old}\left(r\right)=1-\sum_{j=1}^{K+1}\lambda_{s}v_{j}C_{j}^{Old}\left(r\right)$.
5. Solve 4PBik2 Equation $-\nabla \cdot \left(\epsilon_{s}\nabla \phi \left(r\right)\right)+\rho^{\prime }\left(\phi^{Old}\right)\phi \left(r\right)=-\epsilon \Psi^{New}+\rho^{\prime }\left(\phi^{Old}\right)\phi^{Old}$ for $\phi^{New}\left(r\right)$ in $\Omega_{s}$ with $\phi^{New}=V$ on ∂Ω and the same jump condition in Step 2, where $\rho^{\prime }\left(\phi \right)$ is the derivative of ρ(ϕ) with respect to ϕ.
6. If the maximum error norm ${∥\phi^{New}-\phi^{Old}∥}_{\infty }>Tol$, a preset tolerance, then set $\phi^{Old}=\omega_{4PBik}\phi^{Old}+\left(1-\omega_{4PBik}\right)\phi^{New}$ and go to Step 4, else go to Step 7.
7. Solve NP Equation $-\nabla \cdot J_{i}\left(r\right)=0$ for $C_{i}^{New}\left(r\right)$ in $\Omega_{s}$ for all i=1,⋯,K+1 with $J_{i}\left(r\right)=-D_{i}\left[\nabla C_{i}\left(r\right)+\beta_{i}C_{i}\left(r\right)\right]$$\nabla \phi^{Old}\left(r\right)-\lambda_{s}\frac{v_{i}}{v_{0}}C_{i}\left(r\right)\nabla S^{trc}\left(r\right)$, $S^{trc}\left(r\right)=\ln\frac{\Gamma^{Old}\left(r\right)}{\Gamma^{B}}$, $C_{i}^{New}\left(r\right)=0$ on ∂Ω, and $J_{i}\left(r\right)\cdot n=0$ on $\partial \Omega_{m}$.
8. Solve 4PBik1 Equation for $\Psi^{New}$ as in Step 4 with $C_{i}^{New}$ in place of $C_{i}^{Old}$.
9. Solve 4PBik2 Equation for $\phi^{New}$ as in Step 5.
10. If ${∥\phi^{New}-\phi^{Old}∥}_{\infty }>Tol$, then set $\phi^{Old}=\omega_{PNPB}\phi^{Old}+\left(1-\omega_{PNPB}\right)\phi^{New}$ and go to Step 7, else go to Step 11.
11. V=V+ΔV and go to Step 4 until the desired voltage is reached.

Linearizing the nonlinear 4PBik (17) yields two second-order linear 4PBik1 and 4PBik2 in Steps 4 and 5 that differ from the nonlinear (33) and (34). Newton’s iterative Steps 4–6 for solving 4PBik1 and 4PBik2 dictates convergence that also depends on various mappings from an old solution $\phi^{Old}$ to a new solution $\phi^{New}$. This algorithm uses two relaxation and three continuation mappings for which we need to carefully tune two relaxation parameters $\omega_{4PBik}$ and $\omega_{PNPB}$ and three continuation parameters $\lambda_{c}$ (related to correlation effects), $\lambda_{s}$ (steric effects), and ΔV (incremental voltage for applied voltage). For example, the parameter $\lambda_{s}$ in $\Gamma^{Old}\left(r\right)=1-\sum_{j=1}^{K+1}\lambda_{s}v_{j}C_{j}^{Old}\left(r\right)$ can be chosen as $\lambda_{s}=k\Delta \lambda$, $k=0,1,2,\cdots ,\frac{1}{\Delta \lambda }$, an incremental continuation from 0 (no steric effects) to 1 (fully steric effects) with a tuning stepping length Δλ. The algorithm can fail to converge if we choose Δλ=1 (without continuation) for some simulation cases, since we may have $\Gamma^{Old}\left(r\right)<0$ resulting in numerically *undefined*$S^{trc}\left(r\right)=\ln\frac{\Gamma^{Old}\left(r\right)}{\Gamma^{B}}$ at some r where the potential $\phi^{Old}\left(r\right)$ is large.

### 7.2. Discretization Methods

All PDEs in Steps 1, 2, 4, 5, 8, and 9 are of Poisson type $-\nabla^{2}\phi \left(r\right)=f\left(r\right)$. We use the central finite difference (FD) method [64]

(49) $$\frac{-\phi_{i-1,j,k}+2\phi_{ijk}-\phi_{i+1,j,k}}{\Delta x^{2}}+\frac{-\phi_{i,j-1,k}+2\phi_{ijk}-\phi_{i,j+1,k}}{\Delta y^{2}}+\frac{-\phi_{i,j,k-1}+2\phi_{ijk}-\phi_{i,j,k+1}}{\Delta z^{2}}=f_{ijk},$$

to discretize it at all grid points $r_{ijk}=\left(x_{i},y_{j},z_{k}\right)$ in a domain, where $\phi_{ijk}\approx \phi \left(x_{i},y_{j},z_{k}\right)$, $f_{ijk}=f\left(x_{i},y_{j},z_{k}\right)$, and Δx, Δy, and Δz are mesh sizes on the three axes from a uniform partition Δx=Δy=Δz=h. The domains in Steps 1 and 2 are $\Omega_{m}$ and $\Omega_{s}$, respectively. The discretization leads to a sparse matrix system $A\vec{\phi }=\vec{f}$ with the compressed bandwidth of the matrix *A* being 7, where the matrix size can be millions for sufficiently small *h* to obtain sufficiently accurate $\phi_{ijk}$.

The matrix system consists of four subsystems, two by the FD method (49) in $\Omega_{m}$ and $\Omega_{s}$, one by another method (see below) to discretize the jump condition in Step 2 on the interface $\partial \Omega_{m}$ between $\Omega_{s}$ and $\Omega_{m}$, and one by imposing boundary conditions on ∂Ω. We need to solve the matrix system in the entire domain $\bar{\Omega }={\bar{\Omega }}_{m}\cup {\bar{\Omega }}_{s}$.

The convergence order of (49) is $O(h^{2})$ (optimal) in maximum error norm for sufficiently smooth function ϕ(r). However, this optimal order can be easily degraded to $O(h^{0.37})$ [186], for example, by geometric singularities if the jump condition is not properly treated. In [64], we propose the interface method

(50) $$\frac{-\epsilon_{i-\frac{3}{2}}\phi_{i-2}+\left(\epsilon_{i-\frac{3}{2}}+\left(1-A_{1}\right)\epsilon_{i-\frac{1}{2}}^{-}\right)\phi_{i-1}-A_{2}\epsilon_{i-\frac{1}{2}}^{-}\phi_{i}}{\Delta x^{2}}=f_{i-1}+\frac{\epsilon_{i-\frac{1}{2}}^{-}A_{0}}{\Delta x^{2}}$$

(51) $$\frac{-B_{1}\epsilon_{i-\frac{1}{2}}^{+}\phi_{i-1}+\left(\left(1-B_{2}\right)\epsilon_{i-\frac{1}{2}}^{+}+\epsilon_{i+\frac{1}{2}}\right)\phi_{i}-\epsilon_{i+\frac{1}{2}}\phi_{i+1}}{\Delta x^{2}}=f_{i}+\frac{\epsilon_{i-\frac{1}{2}}^{+}B_{0}}{\Delta x^{2}},$$

where

$$A_{1}=\frac{-\left(\epsilon_{m}-\epsilon_{s}\right)}{\epsilon_{m}+\epsilon_{s}},\;A_{2}=\frac{2\epsilon_{m}}{\epsilon_{m}+\epsilon_{s}},\;A_{0}=\frac{-2\epsilon_{m}\left[\phi \right]-\Delta x\left[\epsilon \phi^{\prime }\right]}{\epsilon_{m}+\epsilon_{s}},$$

$$B_{1}=\frac{2\epsilon_{s}}{\epsilon_{m}+\epsilon_{s}},\;B_{2}=\frac{\epsilon_{m}-\epsilon_{s}}{\epsilon_{m}+\epsilon_{s}},\;B_{0}=\frac{2\epsilon_{s}\left[\phi \right]-\Delta x\left[\epsilon \phi^{\prime }\right]}{\epsilon_{m}+\epsilon_{s}},$$

to discretize the 1D Poisson equation $-\frac{d}{dx}\left(\epsilon \left(x\right)\frac{d\phi (x)}{dx}\right)=f\left(x\right)$ at every jump position $\gamma \in \partial \Omega_{m}$ that is at the middle of its two neighboring grid points, i.e., $x_{i-1}<\gamma =x_{i-\frac{1}{2}}<x_{i}$, where $x_{i-\frac{1}{2}}=\left(x_{i-1}+x_{i}\right)/2$ and $x_{i-1}$ and $x_{i}$ belong to different domains $\Omega_{s}$ and $\Omega_{m}$. The corresponding cases in *y*- and *z*-axis follow obviously in a similar way. This method yields *optimal* convergence [64].

Since the matrix system is usually very large in 3D simulations and we need to repeatedly solve such systems updated by nonlinear iterations as shown in the above algorithm, linear iterative methods such as the bi-conjugate gradient stabilized (bi-CG) method are used to solve the matrix system [74]. We propose two parallel algorithms (one for bi-CG and the other for nonlinear iterations) in [74] and show that parallel algorithms on GPU (graphic processing unit) over sequential algorithms on CPU (central processing unit) can achieve 22.8× and 16.9× speedups for the linear solver time and total runtime, respectively.

Discretization of Nernst–Planck equations in Step 7 is different from (49) because the standard FD method

(52) $$\frac{C_{i+1}-C_{i}}{\Delta x}=\frac{C_{i+1}+C_{i}}{2}\left(-\beta \frac{\Delta \phi_{i}}{\Delta x}+\frac{\Delta S_{i}^{trc}}{\Delta x}\right)$$

for the zero flux ($J\left(x\right)=-D\left(x\right)\left(\frac{dC(x)}{dx}+\beta C\left(x\right)\frac{d\phi (x)}{dx}-\frac{v}{v_{0}}C\left(x\right)\frac{dS^{trc}\left(x\right)}{dx}\right)=0$) can easily yield

(53) $$C_{i+1}-C_{i}>C_{i+1}+C_{i}$$

and thereby a negative (*unphysical*) concentration $C_{i}<0$ at $x_{i}$ if

(54) $$\frac{1}{2}\left(-\beta \Delta \phi_{i}+\Delta S_{i}^{trc}\right)>1,$$

where $\Delta \phi_{i-1}=\phi_{i}-\phi_{i-1}$, $\phi_{i}\approx \phi \left(x_{i}\right)$ etc. Therefore, it is crucial to check whether the *generalized Scharfetter–Gummel* (SG) condition [68]

(55) $$-\beta \Delta \phi_{i}+\Delta S_{i}^{trc}\leq 2$$

is satisfied by any discretization method in implementation. This condition generalizes the well-known SG stability condition in semiconductor device simulations [187,188] to include the steric potential function $S^{trc}\left(r\right)$.

We extend the classical SG method [187] of the flux J(x) in [68] to

(56) $$J_{i+\frac{1}{2}}=\frac{-D}{\Delta x}\left[B\left(-t_{i}\right)C_{i+1}-B\left(t_{i}\right)C_{i}\right]$$

where $t_{i}=\beta \Delta \phi_{i}-\Delta S_{i}^{trc}$ and $B\left(t\right)=\frac{t}{e^{t}-1}$ is the Bernoulli function [188]. Equation (56), an exponential fitting scheme, satisfies (55) and is derived from assuming that the flux *J*, the local electric field $\frac{-d\phi }{dx}$, and the local steric field $\frac{dS^{trc}}{dx}$ are all constant in the sufficiently small subinterval $(x_{i}$, $x_{i+1})$, i.e.,

(57) $$\frac{J}{D}=\frac{-dC(x)}{dx}-kC\left(x\right),\;\mathrm{for}\;\mathrm{all}\;x\in \left(x_{i},\;x_{i+1}\right),$$

where $k=\beta \frac{d\phi }{dx}-\frac{dS^{trc}}{dx}$. Solving this ordinary differential equation (ODE) with a boundary condition $C_{i}$ or $C_{i+1}$ yields the well-known Goldman-Hodgkin-Katz flux equation in ion channels [9], which is exactly the same as that in (56) but with the subinterval $(x_{i}$, $x_{i+1})$ being replaced by the height of the entire box in Figure 2R.

The generalized Scharfetter-Gummel method for Nernst-Planck equations is thus

(58) $$\begin{array}{ccc} \frac{dJ(x_{i})}{dx} & \approx & \frac{J_{i+\frac{1}{2}}-J_{i-\frac{1}{2}}}{\Delta x}=\frac{b_{i-1}C_{i-1}+b_{i}C_{i}+b_{i+1}C_{i+1}}{\Delta x^{2}}=0\\ J_{i-\frac{1}{2}} & = & \frac{-D}{\Delta x}\left[B\left(-t_{i-1}\right)C_{i}-B\left(t_{i-1}\right)C_{i-1}\right]\\ J_{i+\frac{1}{2}} & = & \frac{-D}{\Delta x}\left[B\left(-t_{i}\right)C_{i+1}-B\left(t_{i}\right)C_{i}\right]\\ t_{i} & = & \beta \Delta \phi_{i}-\Delta S_{i}^{trc},\;B\left(t\right)=\frac{t}{e^{t}-1}\\ b_{i-1} & = & -B\left(t_{i-1}\right),\;b_{i}=B\left(-t_{i-1}\right)+B\left(t_{i}\right),\;b_{i+1}=-B\left(-t_{i}\right).\; \end{array}$$

The SG method is *optimal* in the sense that it integrates the ODE (57) *exactly* at *every* grid point with a suitable boundary condition [189]. Therefore, the SG method can resolve sharp layers very accurately [189] and hence needs few grid points to obtain tolerable approximations when compared with the primitive FD method. Moreover, the exact solution of (57) for the concentration function C(x) yields an exact flux J(x). Consequently, the SG method is *current preserving*, which is particularly important in nonequilibrium systems, where the current is possibly the most relevant physical property of interest [190].

It is difficult to overstate the importance of the current preserving feature and it must be emphasized for workers coming from fluid mechanics that preserving current has a significance quite beyond the preserving of flux in uncharged systems. Indeed, conservation of current (defined as Maxwell did to include the displacement current of the vacuum $\epsilon_{0}\frac{\partial E(r,t)}{\partial t}$) is an unavoidable consequence, nearly a restatement of the Maxwell equations themselves [104,106]. The electric field is so strong that the tiniest error in preserving current, i.e., the tiniest deviation from Maxwell’s equations, produces huge effects. The third paragraph of Feynman’s lectures on electrodynamics makes this point unforgettable [191]. Thus, the consequences of a seemingly small error in preserving the flow of charge are dramatically larger than the consequences of the same error in preserving the flux of mass.

We have developed a C++ code for solving 4PBik and PNPB models on laptop and high- performance (with GPU) computers. For solving a 4PBik problem with a matrix system of size 4,096,000, for example, the code requires about 300 MB memory to store the compressed matrix system with double precision. It took about 2 min and 47 s on a laptop computer equipped with 1.3 GHz Intel CPU and 2 GB RAM to solve the linear system once by the successive overrelaxation method with an error tolerance of 10${}^{-6}$ [64].

## 8. Applications

We have used the saturating Poisson-Nernst-Planck-Bikerman theory to study ion activities, electric double layers, and biological ion channels in the past. The theory accounts for the steric effect of ions and water molecules, the effects of ion-ion and ion-water correlations, the screening and polarization effects of polar water, and the charge/space competition effect of ions and water molecules of different sizes and valences. These effects are all closely related to the dielectric operator in (17) and the steric potential in (4) that works on both macroscopic and atomic scales. We now illustrate these properties in the following three areas using mostly experimental data to verify the theory.

### 8.1. Ion Activities

**Figure 3:**
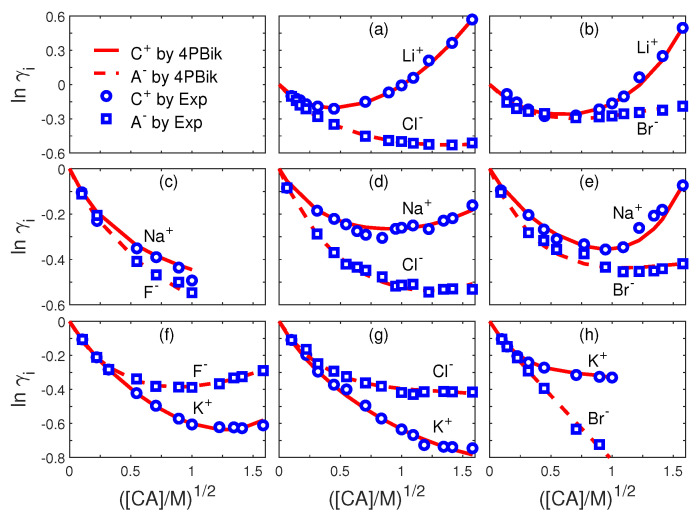
Single-ion activity coefficients of (**a**) LiCl (**b**) LiBr (**c**) NaF (**d**) NaCl (**e**) NaBr (**f**) KF (**g**) KCl (**h**) KBr electrolytes. Comparison of 4PBik results (curves) with experimental data (symbols) [192] on i= C${}^{+}$ (cation) and A${}^{-}$ (anion) activity coefficients $\gamma_{i}$ in various [CA] from 0 to 1.6 M.

The curves in Figure 3 obtained by the generalized Debye-Hückel Formula (45) [75] fit well to the experimental data by Wilczek–Vera et al. [192] for single-ion activities in 8 1:1 electrolytes. There are only three fitting parameters in the formula, namely $\alpha_{1}^{i}$, $\alpha_{2}^{i}$ and $\alpha_{3}^{i}$, which we reiterate have specific physical meaning as parameters of the water shell around ions. The values of the parameters are given in Table 1 from which we observe that $R_{i}^{Born}$ deviates from $R_{i}^{0}$ slightly. For example, $R_{{}_{{\mathrm{Cl}}^{-}}}^{Born}/R_{{\mathrm{Cl}}^{-}}^{0}=1.007∼1.044$ (not shown) for Figure 3a with [LiCl] = 0∼2.5 M, since the cavity radius $R_{{}_{{\mathrm{Cl}}^{-}}}^{Born}$ is an atomic measure from the infinite singularity δ(r−0) at the origin, i.e., $\phi^{4PBik}\left(r\right)$ and thus $\gamma_{i}^{4PBik}$ are very sensitive to $R_{i}^{Born}$. On the other hand, $\gamma_{i}^{4PBik}$ is not very sensitive to $R_{i}^{sh}$ ($R_{{\mathrm{Cl}}^{-}}^{sh}=5.123∼5.083$ Å), i.e., the fixed choice of $O_{i}^{w}=18$ (an experimental value in [166]) for all curves is not critical but reasonable [69]. The error between the estimated $O_{i}^{w}$ and its unknown true value can always be compensated by small adjustments of $R_{i}^{Born}$. The values of other symbols are $a_{{\mathrm{Li}}^{+}}=0.6$ Å, $a_{{\mathrm{Na}}^{+}}=0.95$ Å, $a_{K^{+}}=1.33$ Å, $a_{F^{-}}=1.36$ Å, $a_{{\mathrm{Cl}}^{-}}=1.81$ Å, $a_{{\mathrm{Br}}^{-}}=1.95$, $a_{H_{2}O}=1.4$ Å, $R_{{\mathrm{Li}}^{+}}^{0}=1.3$ Å, $R_{{\mathrm{Na}}^{+}}^{0}=1.618$ Å, $R_{K^{+}}^{0}=1.95$ Å, $R_{F^{-}}^{0}=1.6$ Å, $R_{{\mathrm{Cl}}^{-}}^{0}=2.266$, $R_{{\mathrm{Br}}^{-}}^{0}=2.47$ Å [173], $\epsilon_{w}=78.45$, $\epsilon_{ion}=1$, T=298.15 K, where $a_{i}$ is the (Pauling) radius of type *i* particle (ion) [173].

**Figure 4:**
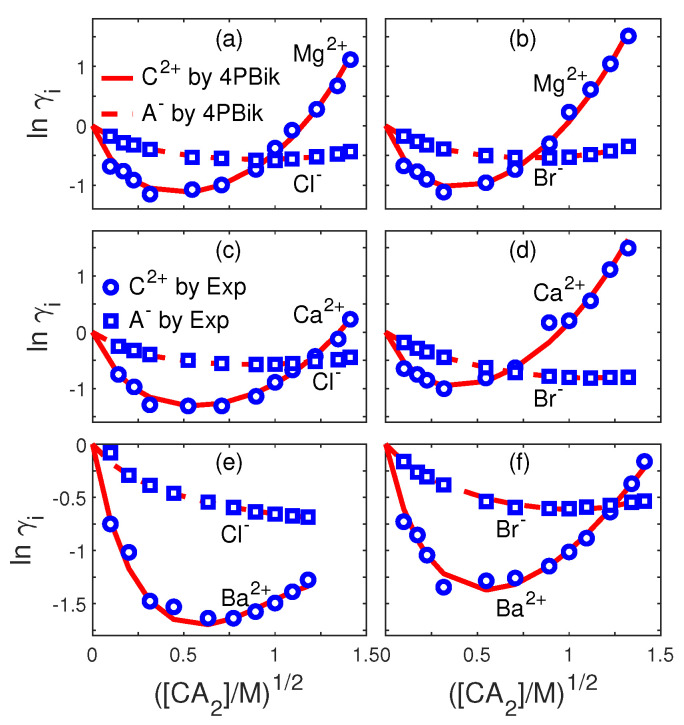
Single-ion activity coefficients of (**a**) MgCl${}_{2}$ (**b**) MgBr${}_{2}$ (**c**) CaCl${}_{2}$ (**d**) CaBr${}_{2}$ (**e**) BaCl${}_{2}$ (**f**) BaBr${}_{2}$ electrolytes. Comparison of 4PBik results (curves) with experimental data (symbols) [192] on i= C${}^{2+}$ (cation) and A${}^{-}$ (anion) activity coefficients $\gamma_{i}$ in various [CA${}_{2}$] from 0 to 1.5 M.

Table 1 also shows the significant order of these parameters, i.e., $\left|\alpha_{1}^{i}\right|>\left|\alpha_{2}^{i}\right|>\left|\alpha_{3}^{i}\right|$ in general cases. Please note that the three sets of the values of $\alpha_{1}^{{\mathrm{Na}}^{+}}$, $\alpha_{2}^{{\mathrm{Na}}^{+}}$, and $\alpha_{3}^{{\mathrm{Na}}^{+}}$ for the same Na${}^{+}$ in three different salts NaCl, NaBr, and NaF are different because the diameters of the anions are different. Figure 4 shows single-ion activities in 6 2:1 electrolytes by experiments [192] and 4PBik, where the significant order (not shown) of three fitting parameters is similar to that in Table 1.

**Figure 5:**
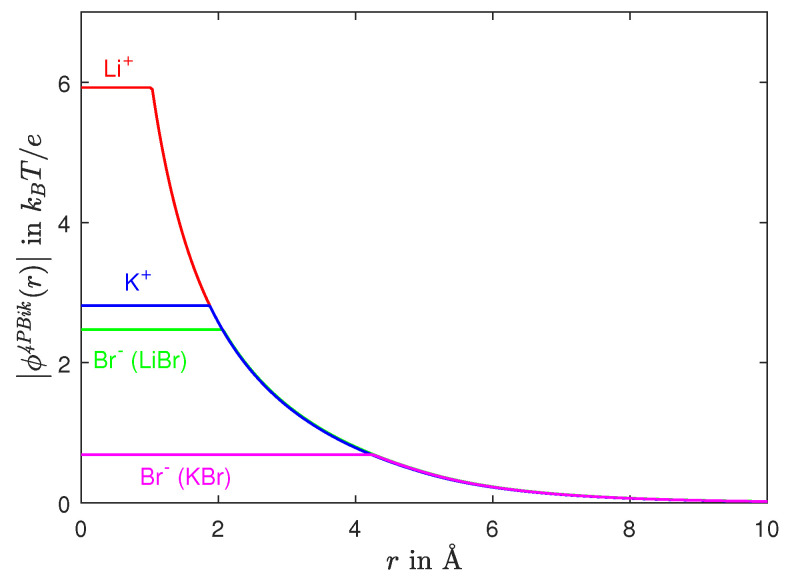
Electric potential $\phi^{4PBik}\left(r\right)$ profiles by (42) near the solvated ions Li${}^{+}$ and Br${}^{-}$ at [LiBr] = 2 M, and K${}^{+}$ and Br${}^{-}$ at [KBr] = 2 M, where *r* is the distance from the center of the respective ion.

The electric potential and other physical properties of ionic activity can be studied in detail according to the partitioned domain in Figure 1 characterized by $R_{i}^{Born}$ and $R_{i}^{sh}$. For example, we observe from Figure 5 that the electric potential ($\phi_{{\mathrm{Br}}^{-}}^{4PBik}\left(0\right)=-2.4744$$k_{B}T/e$) and the Born radius ($R_{{\mathrm{Br}}^{-}}^{Born}($2 M)=2.0637 Å) generated by Br${}^{-}$ at [LiBr] = 2 M are significantly different from that ($\phi_{{\mathrm{Br}}^{-}}^{4PBik}\left(0\right)=-0.6860$ $k_{B}T/e$, $R_{{\mathrm{Br}}^{-}}^{Born}($2 M)=4.2578 Å) at [KBr] = 2 M. The only difference between these two solutions is the size of cations, i.e., the size of different positive ions significantly changes the activity of the same negative ion at high concentrations. The difference between $\phi_{{\mathrm{Li}}^{+}}^{4PBik}\left(0\right)$ and $\phi_{K^{+}}^{4PBik}\left(0\right)$ is due to the sizes of Li${}^{+}$ and K${}^{+}$ not Br${}^{-}$ as it is the same for both solutions.

This example clearly shows the atomic properties of 4PBik theory in the ion $\Omega_{i}$ and shell $\Omega_{sh}$ domains and the continuum properties in the solvent domain $\Omega_{s}$. The Born radius $R_{i}^{Born}$ in (42) determined by (44) changes with (i) *ion-water* interactions in $\Omega_{i}\cup \Omega_{sh}$ and (ii) *ion-ion* interactions in $\Omega_{i}\cup \Omega_{s}$ via $\phi^{4PBik}\left(r\right)$ in (42) that is self-consistently determined by the interface conditions in (35)–(39) and by (iii) *multi-salt* [73,76] concentrations in $\Omega_{s}$, (iv) the *screening* effects of water in $\Omega_{sh}$ and ions and water in $\Omega_{s}$, (v) the *polarization* effect of water in $\Omega_{s}$, (vi) the *correlation* effect between ions in $\Omega_{s}$, (vii) the *steric* effects of all ions and water in the entire domain $\bar{\Omega }={\bar{\Omega }}_{i}\cup {\bar{\Omega }}_{sh}\cup {\bar{\Omega }}_{s}$, (viii) *temperatures* [73,76], and (ix) *pressures* [73,76]. The generalized Debye-Hückel formula (45) includes all these 9 physical properties with only 3 fitting parameters. However, we look forward to the day when we can derive the three fitting parameters for particular types of ions, from independently determined experimental data.

### 8.2. Electric Double Layers

We consider a charged surface in contact with a 0.1 M 1:4 aqueous electrolyte, where the charge density is σ=1e/(50Å${}^{2})$, the radius of both cations and anions is a=4.65 Å (in contrast to an edge length of 7.5 Å of cubical ions in [133]), and $\epsilon_{s}=80$ [72]. The multivalent ions represent large polyanions adsorbed onto a charged Langmuir monolayer in experiments [133]. We solve (33) and (34) using (49) in the rectangular box $\bar{\Omega }={\bar{\Omega }}_{s}=\left\{(x,y,z):0\leq x\leq 40,\;-5\leq y\leq 5,\;-5\leq z\leq 5\;Å\right\}$ such that ϕ(r)≈0 within the accuracy to $10^{-4}$ near and on the surface x=40 Å. The boundary conditions on the surface and its adjacent four planes are $-\epsilon_{s}\nabla \phi \cdot n=\sigma$ with $n=\langle -1,0,0\rangle$ and $-\epsilon_{s}\nabla \phi \cdot n=0$ with n defined similarly, respectively.

**Figure 6:**
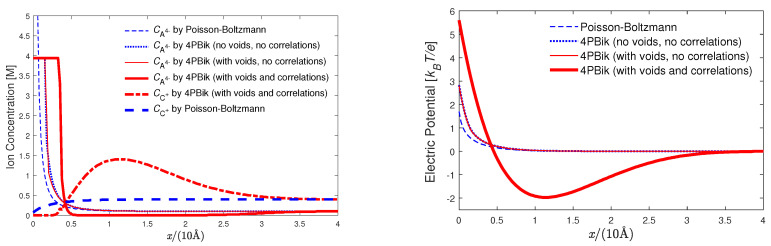
**Left (L)**: Concentration profiles of anions $C_{A^{4-}}\left(x\right)$ and cations $C_{C^{+}}\left(x\right)$ obtained by various models in a C${}_{4}$A electrolyte solution with the charge density σ=1e/(50Å${}^{2})$ at x=0. **Right (R)**: Electric potential profiles ϕ(x).

The classical PB model (with $a=a_{H_{2}O}=l_{c}=0$, i.e., no size, void, and correlation effects) produces unphysically high concentrations of anions (A${}^{4-}$) near the surface as shown by the dashed curve in Figure 6L. The dotted curve in Figure 6L is similar to that of the modified PB in [133] and is obtained by the 4PBik model with $l_{c}=0$ (no correlations), $V_{K+2}=0$ (no voids), and $a_{H_{2}O}=0$ (water is volumeless as in [133] and hence $\Gamma^{B}=1-\sum_{i=1}^{K}v_{i}C_{i}^{B}$ is the bulk water volume fraction). The voids ($V_{K+2}\neq 0$) and water molecules ($a_{H_{2}O}\neq 0$) have slight effects on anion concentration (because of saturation) and electric potential (because water and voids have no charges) profiles as shown by the thin solid curves in Figure 6L,R, respectively, when compared with the dotted curves. However, ion-ion correlations (with $l_{c}=1.6a$ [78]) have significant effects on ion distributions as shown by the thick solid and dash-dotted curves in Figure 6L, where the saturation layer is on the order of ionic radius *a* and the *overscreening* layer [78] ($C_{A^{4-}}\left(x\right)\approx 0<C_{A^{4-}}^{B}=0.1$ M) of excess co-ions ($C_{C^{+}}\left(x\right)>C_{C^{+}}^{B}=0.4$ M) is about 18 Å in thickness.

The *saturation layer* is an *output* (not an imposed condition) of our model unlike a Stern layer [193] imposed by most EDL models to account for size effects near charge surfaces [194,195,196]. The electric potentials ϕ(0)= 5.6 $k_{B}T/e$ at x=0 and ϕ(11.5)=−1.97 $k_{B}T/e$ in Figure 6R obtained by 4PBik with voids and correlations deviate dramatically from those by previous models for nearly 100% at x=0 (in the saturation layer) and 70% at x=11.5 Å (in the screening layer) when compared with the maximum potential ϕ(0)=2.82 $k_{B}T/e$ of previous models. The 4PBik potential depth ϕ(11.5)=−1.97 $k_{B}T/e$ of the overscreening layer is very sensitive the size *a* of ions and tends to zero as a→0.

### 8.3. Biological Ion Channels

Biological ion channels are a particularly appropriate test of a model of concentrated ionic solutions.

The data available for tens to hundreds of different types of channels and transporters is breathtaking: it is often accurate to a few per cent (because signal to noise ratios are so large and biological variation hardly exists for channels of known amino acid sequence, which means nearly every channel presently). The data is always nonequilibrium, i.e., current voltage relations in a wide range of solutions of different composition and concentration, or (limiting zero voltage) conductance in those solutions. Indeed, many of the channels do not function if concentrations are equal on both sides and the electrical potential is zero. They are said to inactivate.

The data is often available for single channels recorded individually in patch clamp or bilayer configuration. Data is available for a range of divalent (usually calcium ion) concentrations because calcium concentration is often a controller of channel, transporter, and biological activity in the same sense that a gas pedal is the controller of the speed of a car. The structure of the ion channel or transporter is often known in breathtaking detail. The word ‘breathtaking’ is appropriate because similar structures are rarely if ever known of strictly physical systems. The structure and the structure of the permanent and polarization charge of the channel protein (that forms the pore through which ions move) can be modified by standard methods of site directed mutagenesis, for example that are available in ‘kit’ form usable by most molecular biology laboratories. Thus, models can be tested from atomic detail to single-channel function to ensemble function to cellular and physiological function, even to the ultimate biological function (like the rate of the heartbeat). Few other systems allow experimental measurement at each level of the hierarchy linking the atomic composition of genes (that encode the channel’s amino acid composition), to the atomic structure of the channel, right to the function of the cell. The hierarchy here reaches from 10${}^{-11}$ to 10${}^{-5}$ m. When the channel controls the biological function of an organ like the heart, the hierarchy reaches to $2\cdot 10^{-1}$ m, in humans for example.

The biological significance of ion channels is hard to exaggerate since they play a role in organisms analogous to the role of transistors in computers. They are the device that execute most of the physical controls of current and ion movement that are then combined in a hierarchy of structures to make biological cells, tissues, and organisms, if not populations of organisms.

From a physical point of view, ion channels provide a particularly crowded environment in which the effects of the steric potential (crowding in more traditional language) and electrical potential can combine to produce striking characteristics of selectivity and rectification. Theories that do not deal explicitly with ion channel data, i.e., that do not predict current voltage relations from known structures, seem to us to be begging central PHYSICAL questions that might falsify their approach. In fact, as a matter of history it is a fact that we learned how to construct our model of bulk solutions from our earlier work on ion channels.

#### 8.3.1. Gramicidin A Channel

We use the gramicidin A (GA) channel in Figure 2L to illustrate the full Poisson–Nernst–Planck–Bikerman theory consisting of Equations (4), (27), (28), (32)–(34), and conditions (35)–(39) with—steric, correlation, polarization, dielectric, charge/space competition, and nonequilibrium effects—at steady state using the algorithm and methods in Section 7 to perform numerical simulations. The union domain ${\bar{\Omega }}_{i}\cup \Omega_{sh}$ in Figure 1 is replaced by the biomolecular domain $\Omega_{m}$ in Figure 2R.

**Figure 7:**
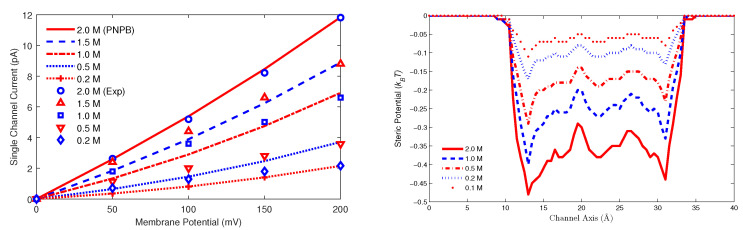
**Left (L)**: A comparison of PNPB (curves) and experimental [197] (symbols) I-V results with bath K${}^{+}$ and Cl${}^{-}$ concentrations $C^{B}=0.1$, 0.2, 0.5, 1, 2 M and membrane potentials ΔV=0, 50, 100, 150, 200 mV. **Right (R)**: Averaged steric potential $S^{trc}\left(r\right)$ profiles at each cross section along the pore axis with $C^{B}=$ 0.1, 0.2, 0.5, 1, 2 M and ΔV=200 mV. The same averaging method applies to the following profiles.

Figure 7L shows I-V curves obtained by PNPB and compared with experimental data (symbols) by Cole et al. [197] with bath K${}^{+}$ and Cl${}^{-}$ concentrations $C^{B}=0.1$, 0.2, 0.5, 1, 2 M and membrane potentials ΔV=0, 50, 100, 150, 200 mV. The PNPB currents in pico ampere (pA) were obtained with θ=1/4.7 in the pore diffusion coefficients $\theta D_{i}$ from (30) for all particle species. The reduction parameter θ has been used in all previous PNP papers and is necessary for continuum is comparable to MD, BD, or experimental data [198]. The values of other model parameters are listed in Table I in [68].

**Figure 8:**
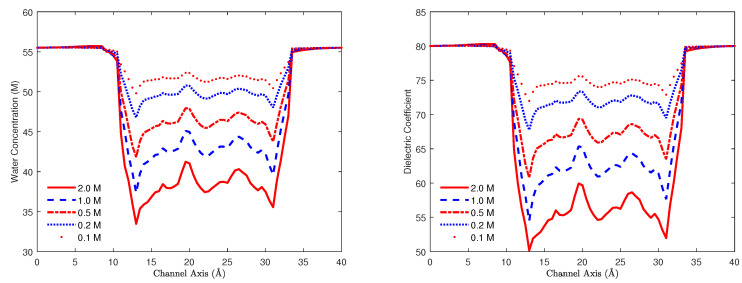
**Left (L)**: Water concentration $C_{H_{2}O}\left(r\right)$ profiles. **Right (R)**: Dielectric function $\tilde{\epsilon }\left(r\right)$ profiles.

**Figure 9:**
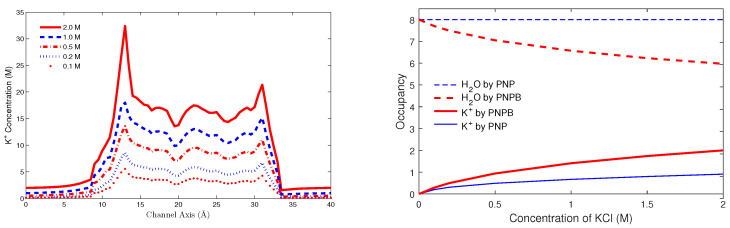
**Left (L)**: K${}^{+}$ concentration $C_{K^{+}}\left(r\right)$ profiles. **Right (R)**: Occupancy of H${}_{2}$O and K${}^{+}$ in the GA channel pore by PNPB and PNP as [KCl] increases from 0 to 2 M. The total number of H${}_{2}$O and K${}^{+}$ in the pore is 8 [211], which is conserved by PNPB but not by PNP (without steric and correlation effects).

We summarize the novel results of PNPB in [68] when compared with those of earlier continuum models for ion channels: (i) The pore *diffusion* parameter θ=1/4.7 agrees with the range 1/3 to 1/10 obtained by many MD simulations of various channel models [199,200,201] indicating that the steric (Figure 7R), correlation, dehydration (Figure 8L), and dielectric (Figure 8R) properties have made PNPB simulations closer (realistic) to MD than previous PNP for which θ differs from MD values by an order to several orders of magnitude [200]. (ii) Figure 7R and Figure 8L,R, which are all absent in earlier work, show that these properties *correlate* to each other and *vary* with salt concentration and protein charges in a *self-consistent* way by PNPB. (iii) The steric potential profiles in Figure 7R clearly illustrate the *charge/space competition* between ions and water under dynamic and variable conditions. For example, the global minimum value in Figure 7R at $\hat{r}=13.1$ on the channel axis, where the channel protein is most negatively charged, is $S^{trc}\left(\hat{r}\right)=\ln\frac{\Gamma (\hat{r})}{\Gamma^{B}}=-0.485$ yielding $\Gamma \left(\hat{r}\right)/\Gamma^{B}=0.616$. Namely it is 38.4% more crowded at $\hat{r}$ than in the bath and mainly occupied by K${}^{+}$ as shown in Figure 8L and Figure 9L. It is important to *quantify voids* ($\Gamma \left(r\right)=1-\sum_{i=1}^{K+1}v_{i}C_{i}\left(r\right)$) at highly charged locations in channel proteins and many more biological, chemical, and nano systems. The charge space competition has been a central topic in the study of ion channels since at least [202,203,204,205,206]. The literature is too large to describe in detail here. Recent reviews can help [207,208,209,210]. (iv) PNPB preserves *mass conservation* due to void and size effects in contrast to PNP as shown in Figure 9R, where the total number of H${}_{2}$O and K${}^{+}$ in the channel pore is 8 [211].

#### 8.3.2. L-Type Calcium Channel

**Figure 10:**
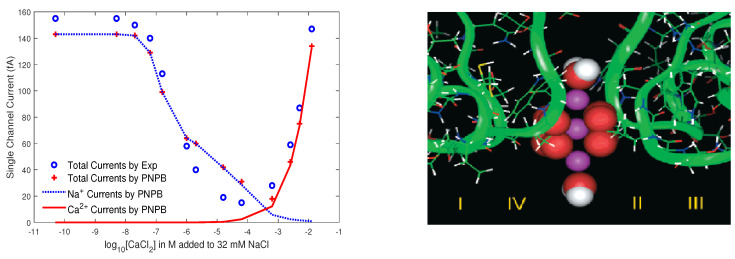
**Left (L)**: Single-channel currents in femto ampere (fA) plotted as a function of $\log_{10}$[Ca${}^{2+}$]${}_{o}$. Experimental data of [212] are marked by small circles and PNPB data are denoted by the plus sign and lines. **Right (R)**: The Lipkind–Fozzard pore model of L-type calcium channel, where 3 Ca${}^{2+}$ are shown in violet, 8 O${}^{1/2-}$ in red, 2 H${}_{2}$O in white and red. Reprinted with permission from (G. M. Lipkind and H. A. Fozzard, Biochem. 40, 6786 (2001)). Copyright (2001) American Chemical Society.

L-type calcium channels operate very delicately in physiological and experimental conditions. They exquisitely tune their conductance from Na${}^{+}$-flow, to Na${}^{+}$-blockage, and to Ca${}^{2+}$-flow when bath Ca${}^{2+}$ varies from trace to high concentrations as shown by the single-channel currents in femto ampere in Figure 10L (circle symbols) recorded by Almers and McCleskey [212], where the range of extracellular concentrations [Ca${}^{2+}$]${}_{o}$ is 10${}^{8}$-fold from $10^{-10}$ to $10^{-2}$ M.

We used the Lipkind-Fozzard molecular model [213] shown in Figure 10R to perform PNPB simulations with both atomic and continuum methods (Algorithm 2 in [68]) for this model channel, where the EEEE locus (four glutamate side chains modeled by 8 O${}^{1/2-}$ ions shown by red spheres) forms a high-affinity Ca${}^{2+}$ binding site (center violet sphere) that is essential to Ca${}^{2+}$ selectivity, blockage, and permeation. Water molecules are shown in white and red. More realistic structures would be appropriate if the work were done now, but the analysis here shows the ability of PNPB to deal with experimental data using even a quite primitive model of the structure.

PNPB results (plus symbols) in Figure 10L agree with the experimental data at [${\mathrm{Na}}^{+}$]${}_{i}=$ [${\mathrm{Na}}^{+}$]${}_{o}=32$ mM, [Ca${}^{2+}$]${}_{i}=0$, $V_{o}=0$, and $V_{i}$=−20 mV (the intracellular membrane potential), where the partial Ca${}^{2+}$ and Na${}^{+}$ currents are denoted by the solid and dotted line, respectively. These two ionic currents show the anomalous mole fraction effect of the channel at nonequilibrium, i.e., trace concentrations of Ca${}^{2+}$ ions effectively block the flow of abundant monovalent cations [212].

#### 8.3.3. Potassium Channel

**Figure 11:**
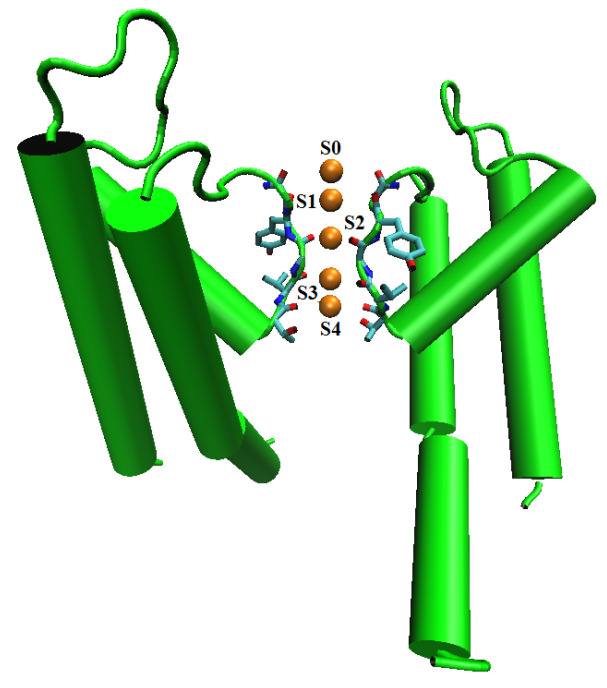
The crystal structure of the K channel KcsA (PDB ID 3F5W) [214] with five cation binding sites S0, S1, S2, S3, and S4 [215] marked by spheres.

Potassium channels conduct K${}^{+}$ ions very rapidly (nearly at the diffusion rate limit (10${}^{8}$ per second) in bulk water) and selectively (excluding, most notably, Na${}^{+}$ despite their difference in radius is only $a_{K^{+}}-a_{{\mathrm{Na}}^{+}}=1.33-0.95=0.38$ Å in sub-Angstrom range) [9]. Figure 11 shows the structure of KcsA (PDB ID 3F5W) crystallized by Cuello et al. [214], where the spheres denote five specific cation binding sites (S0 to S4) [215] in the solvent domain $\Omega_{s}$ and the channel protein in $\Omega_{m}$ consists of *N* = 31,268 charged atoms. The exquisite selectivity of K${}^{+}$ over Na${}^{+}$ by K channels can be quantified by the free energy (*G*) differences of K${}^{+}$ and Na${}^{+}$ in the pore and in the bulk solution, i.e., by ΔG(K${}^{+})=\left[G_{\mathrm{pore}}\left(K^{+}\right)-G_{\mathrm{bulk}}\left(K^{+}\right)\right]$ and ΔG(Na${}^{+})=G_{\mathrm{pore}}($Na${}^{+})-G_{\mathrm{bulk}}($Na${}^{+})$ [215]. Experimental measurements [216,217,218] showed that the relative free energy

(59) $$\Delta \Delta G\left(K^{+}\to {\mathrm{Na}}^{+}\right)=\Delta G\left({\mathrm{Na}}^{+}\right)-\Delta G\left(K^{+}\right)=5∼6\;\mathrm{kcal}/\mathrm{mol}$$

unfavorable for Na${}^{+}$.

Free energies can be calculated by the electric and steric potentials [72]

(60) $$\phi_{S2}=\frac{1}{4\pi \epsilon_{0}}\left(\frac{1}{6}\sum_{k=1}^{6}\sum_{j=1}^{N}\frac{q_{j}}{\epsilon_{p}\left(r\right)\left|c_{j}-A_{k}\right|}+\frac{q_{S2}}{\epsilon_{b}a_{S2}}\right),\;S_{S2}^{trc}=\ln\frac{1-\frac{v_{S2}}{V_{S2}}}{\Gamma^{B}},$$

at the binding site S2 [215] on the atomic scale, where S2 also denotes Na${}^{+}$ or K${}^{+}$ (the site is occupied by a Na${}^{+}$ or K${}^{+}$), $q_{j}$ is the charge on the atom *j* in the protein given by PDB2PQR [219], $\epsilon_{p}\left(r\right)=1+77r/\left(27.7+r\right)$ [119], $r=|c_{j}-$$c_{S2}|$, $c_{j}$ is the center of atom *j*, $A_{k}$ is one of six symmetric surface points on the spherical S2, $\epsilon_{b}=3.6$, and $V_{S2}=1.5v_{K^{+}}$ is a volume containing the ion at S2. We obtained ΔΔG=5.26 kcal/mol [72] in accord with the MD result 5.3 kcal/mol [215], where $G_{\mathrm{pore}}($Na${}^{+})=4.4$, $G_{\mathrm{bulk}}($Na${}^{+})=-0.26$, $G_{\mathrm{pore}}($K${}^{+})=-0.87$, $G_{\mathrm{bulk}}($K${}^{+})=-0.27$ kcal/mol, $\phi_{{\mathrm{Na}}^{+}}=7.5$$k_{B}T/e$, $\frac{v_{{\mathrm{Na}}^{+}}}{v_{0}}S_{{\mathrm{Na}}^{+}}^{trc}=0.23$, $\phi_{K^{+}}=-1.93$$k_{B}T/e$, $\frac{v_{K^{+}}}{v_{0}}S_{K^{+}}^{trc}=-0.59$, and $C_{{\mathrm{Na}}^{+}}^{B}=C_{K^{+}}^{B}=0.4$ M.

The crucial parameter in (60) is the ionic radius $a_{S2}=0.95$ or 1.33 Å (also in $|c_{j}-A_{k}|$) that affects $\phi_{S2}$ very strongly but $S_{S2}^{trc}$ weakly. Another important parameter in (60) is the bulk void fraction $\Gamma^{B}$ that depends on the bulk concentrations of all ions and water and links the total energy of the ion at S2 to these bulk conditions measured very far away (∼10${}^{6}$ Å) in the baths on the atomic scale.

#### 8.3.4. Sodium Calcium Exchanger

**Figure 12:**
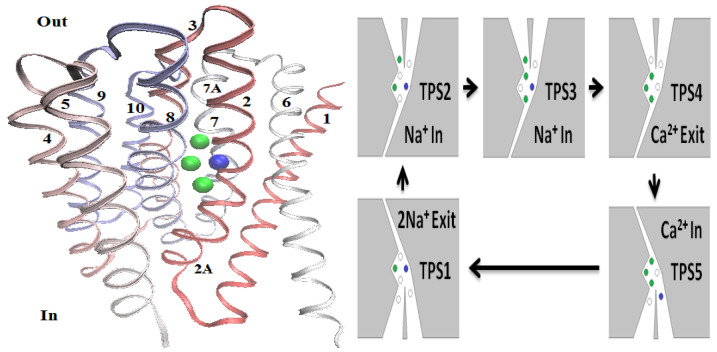
**Left (L)**: Structure of NCX_Mj consisting of ten transmembrane helices that form a binding pocket of three Na${}^{+}$ (green spheres) and one Ca${}^{2+}$ (blue sphere) binding sites [222]. **Right (R)**: Schematic diagram of a cycle of Na${}^{+}$/Ca${}^{2+}$ exchange in NCX consisting of five total potential states (TPS). Two Na${}^{+}$ and one Ca${}^{2+}$ ions enter the binding pocket in the outward- (TPS2 → TPS3 → TPS4) and inward-facing (TPS5 → TPS1) conformations, respectively. They exit in opposite conformations [70].

The Na${}^{+}$/Ca${}^{2+}$ exchanger (NCX) is the major cardiac mechanism that extrudes intracellular Ca${}^{2+}$ across the cell membrane against its chemical gradient by using the downhill gradient of Na${}^{+}$ [28]. The molecular basis of Na${}^{+}$/Ca${}^{2+}$ interactions in NCX so striking to Lüttgau and Niedegerke [220] have been revealed by the cloning of NCX gene [221] and the structure of the ancient archaebacterial version NCX_Mj determined by Liao et al. [222]. Figure 12L illustrates NCX_Mj that consists of 10 transmembrane (TM) helices in which eight helices (TMs 2 to 5 and 7 to 10 labeled numerically in the figure) form a binding pocket of three putative Na${}^{+}$ (green spheres) and one Ca${}^{2+}$ (blue sphere) binding sites [222].

We developed a cyclic model of Na${}^{+}$/Ca${}^{2+}$ exchange mechanism in NCX [70] using (60) to calculate five total (electric and steric) potential states (TPS) of various Na${}^{+}$ and Ca${}^{2+}$ ions shown in Figure 12R, where TPS1 and TPS4 are stable (with negative values) and TPS2, TPS3, and TPS5 are unstable (positive). Four extra sites in Figure 12R are determined empirically and close to entrance or exit locations of the binding pocket. The green and blue dots in the diagram represent Na${}^{+}$ and Ca${}^{2+}$ ions occupying the respective sites. Two Na${}^{+}$ and one Ca${}^{2+}$ ions enter the binding pocket in the outward- (TPS2 → TPS3 → TPS4) and inward-facing (TPS5 → TPS1) conformations, respectively. They exit in opposite conformations. The cycle consists of five steps.

*Step 1:* A conformational change is hypothetically activated [70] by a binding Ca${}^{2+}$ at the blue site (S1) in TPS1 from inward-facing to outward-facing in TPS2.

*Step 2:* One Na${}^{+}$ enters the binding pocket from the access site in TPS2 to the top Na${}^{+}$ binding site (S2) in TPS3 followed by another Na${}^{+}$ to the access site. These two coming Na${}^{+}$ ions move the existing Na${}^{+}$ ion from the middle Na${}^{+}$ site (S3) to the bottom site (S4) by their Coulomb forces. TPS2 and TPS3 are unstable meaning that the two coming Na${}^{+}$ ions have positive energies and are thus mobile. The selectivity ratio of Na${}^{+}$ to Ca${}^{2+}$ by NCX from the extracellular bath to the binding site S2 is $C_{{\mathrm{Na}}^{+}}($S2$)/C_{{\mathrm{Ca}}^{2+}}\left(S2\right)=55.4$ under the experimental conditions of the extracellular bath ${\left[{\mathrm{Na}}^{+}\right]}_{o}=120$ mM and ${\left[{\mathrm{Ca}}^{2+}\right]}_{o}=1$μM [70].

*Step 3:* The vacant site S3 in TPS3 is a deep potential well with TP = −8.89$k_{B}T/e$ that pulls the two unstable Na${}^{+}$ ions to their sites in TPS4. Meanwhile, these two moving Na${}^{+}$ and the stable Na${}^{+}$ at S4 extrude the Ca${}^{2+}$ (with unstable TP = 1.65) at S1 out of the pocket to become TPS4.

*Step 4:* Now, all three Na${}^{+}$ ions in TPS4 are stable with negative TP and the vacant site S1 has an even deeper TP = −16.02. We conjecture that this TP value may trigger a conformational change from outward-facing in TPS4 to inward-facing in TPS5. The mechanism of conformational changes in NCX is yet to be studied.

*Step 5:* Furthermore, this large negative TP in TPS5 yields a remarkably large *selectivity* ratio of Ca${}^{2+}$ to Na${}^{+}$ by NCX from the intracellular bath to S1, i.e., $C_{{\mathrm{Ca}}^{2+}}($S1$)/C_{{\mathrm{Na}}^{+}}($S1)=4986.1 at ${\left[{\mathrm{Ca}}^{2+}\right]}_{i}=33$μM and ${\left[{\mathrm{Na}}^{+}\right]}_{i}=60$ mM. A coming Ca${}^{2+}$ in TPS5 then extrudes two Na${}^{+}$ ions out of the packet when it settles at S1 in stable TPS1.

Assuming that the total time T of an exchange cycle is equally shared by the 5 TPS, this model also infers that the stoichiometry of NCX is $\frac{3}{5}$T·2 Na${}^{+}:\frac{2}{5}$T·1 Ca${}^{2+}=3$ Na${}^{+}:1$ Ca${}^{2+}$ in transporting Na${}^{+}$ and Ca${}^{2+}$ ions [70], which is the generally accepted stoichiometry (see reviews of Blaustein and Lederer [223] and Dipolo and Beaugé [224]) since the pivotal work of Reeves and Hale [225] and other subsequent experimental results. Please note that our model does not consider the electrogenic property of NCX [223], i.e., the driving force of the electric potential gradient.

## 9. Conclusions

We have covered a range of aspects of the fourth-order Poisson-Nernst-Planck-Bikerman theory from physical modeling, mathematical analysis, numerical implementation, to applications and verifications for aqueous electrolyte systems in chemistry and biology. The theory can describe many properties of ions and water in the system that classical theories fail to describe such as steric, correlation, polarization, variable permittivity, dehydration, mass conservation, charge/space competition, overscreening, selectivity, saturation, and more. All these properties are accounted for in a single framework with only two fundamental parameters, namely the dielectric constant of pure water and the correlation length of empirical choice. Ions and water have their physical volumes as those in molecular dynamic simulations. The theory applies to a system at both continuum and atomic scales due to the exact definition of the total volume of all ions, water molecules, and interstitial voids.

The most important features of PNPB are that (i) ions and water have unequal volumes with interstitial voids, (ii) their distributions are saturating of the Fermi type, (iii) these Fermi distributions approach Boltzmann distributions as the volumes tend to zero, and (iv) all the above physical properties appear self-consistently in a single model not separately by various models. Most existing modified Poisson-Boltzmann models consider ions of equal size and fail to yield Boltzmann distributions in limiting cases, i.e., the limit is divergent indicating that steric energies are poorly estimated. Numerous models for different properties such as steric, correlation, polarization, permittivity are proposed separately in the past.

We have shown how to solve 4PBik analytically and PNPB numerically. The generalized Debye-Hückel theory derived from the 4PBik model gives valuable insights into physical properties and leads to an electrolyte (analytical) equation of state that is useful to study thermodynamic activities of ion and water under wide ranges of composition, concentration, temperature, and pressure.

Numerically solving the fourth-order PNPB model in 3D for realistic problems is a challenging task. There are many pitfalls that one must carefully avoid in coding. For that reason, we have particularly mentioned some methods for handling the convergence issues of the highly nonlinear PNPB system of partial differential equations and the discretization problems concerning the complicate interface between molecular and solvent domains and the Scharfetter-Gummel stability condition to ensure positivity of numerical concentrations and current preservation.

Finally, we have shown novel results obtained by PNPB for chemical and biological systems on ion activities, electric double layers, gramicidin A channel, L-type calcium channel, potassium channel, and sodium calcium exchanger. These results agree with experiments or molecular dynamics data and show not only continuum but also atomic properties of the system under far-field conditions. The fourth-order PNPB model is consistent and applicable to a great variety of systems on a vast scale from *meter* to *Angstrom*.

## Figures and Tables

**Table 1:** Values of $\alpha_{1}^{i}$, $\alpha_{2}^{i}$, $\alpha_{3}^{i}$ in (44).

Fig.#	*i*	$\alpha_{1}^{i}$	$\alpha_{2}^{i}$	$\alpha_{3}^{i}$	Fig.#	*i*	$\alpha_{1}^{i}$	$\alpha_{2}^{i}$	$\alpha_{3}^{i}$
3a	Li${}^{+}$	−0.006	−0.037	0.004	3e	Na${}^{+}$	−0.049	0.042	−0.013
3a	Cl${}^{-}$	0.052	−0.015	0	3e	Br${}^{-}$	0.071	−0.048	0.006
3b	Li${}^{+}$	−0.006	−0.011	−0.004	3f	K${}^{+}$	0.005	0.051	−0.015
3b	Br${}^{-}$	0.026	−0.057	0.010	3f	F${}^{-}$	0.033	−0.028	0.003
3c	Na${}^{+}$	0	0	0	3g	K${}^{+}$	0.031	0.022	−0.005
3c	F${}^{-}$	0.027	0	0	3g	Cl${}^{-}$	0.020	−0.025	0.004
3d	Na${}^{+}$	−0.045	0.009	−0.002	3h	K${}^{+}$	0.025	−0.062	0.018
3d	Cl${}^{-}$	0.063	−0.014	−0.002	3h	Br${}^{-}$	0.001	0.082	0

## Abbreviations

The following abbreviations are used in this manuscript:

4PBik	Fourth-Order Poisson-Bikerman
BD	Brownian Dynamics
bi-CG	bi-Conjugate Gradient Stabilized
DH	Debye-Hückel
EDL	Electric Double-Layer
FD	Finite Difference
GA	Gramicidin A
JESS	Joint Expert Speciation System
L-J	Lennard–Jones
MC	Monte Carlo
MD	Molecular Dynamics
NCX	Sodium Calcium Exchanger
NP	Nernst-Planck
ODE	Ordinary Differential Equation
OZ	Ornstein-Zernike
PB	Poisson–Boltzmann
PDE	Partial Differential Equation
PDB	Protein Data Bank
PNP	Poisson-Nernst-Planck
PNPB	Poisson-Nernst-Planck-Bikerman
SG	Scharfetter-Gummel
TPS	Total Potential State
